# In the Absence of Central pre-B Cell Receptor Selection, Peripheral Selection Attempts to Optimize the Antibody Repertoire by Enriching for CDR-H3 Y101

**DOI:** 10.3389/fimmu.2018.00120

**Published:** 2018-02-07

**Authors:** Mohamed Khass, Tessa Blackburn, Ada Elgavish, Peter D. Burrows, Harry W. Schroeder

**Affiliations:** ^1^Department of Medicine, University of Alabama at Birmingham, Birmingham, AL, United States; ^2^Genetic Engineering and Biotechnology Division, National Research Center, Cairo, Egypt; ^3^Department of Microbiology, University of Alabama at Birmingham, Birmingham, AL, United States

**Keywords:** antibody repertoire, B cell development, preBCR, CDR-H3, peripheral B cell subsets, BrdU incorporation, apoptosis

## Abstract

Sequential developmental checkpoints are used to “optimize” the B cell antigen receptor repertoire by minimizing production of autoreactive or useless immunoglobulins and enriching for potentially protective antibodies. The first and apparently most impactful checkpoint requires μHC to form a functional pre-B cell receptor (preBCR) by associating with surrogate light chain, which is composed of VpreB and λ5. Absence of any of the preBCR components causes a block in B cell development that is characterized by severe immature B cell lymphopenia. Previously, we showed that preBCR controls the amino acid content of the third complementary determining region of the H chain (CDR-H3) by using a VpreB amino acid motif (RDR) to select for tyrosine at CDR-H3 position 101 (Y101). In antibodies bound to antigen, Y101 is commonly in direct contact with the antigen, thus preBCR selection impacts the antigen binding characteristics of the repertoire. In this work, we sought to determine the forces that shape the peripheral B cell repertoire when it is denied preBCR selection. Using bromodeoxyuridine incorporation and evaluation of apoptosis, we found that in the absence of preBCR there is increased turnover of B cells due to increased apoptosis. CDR-H3 sequencing revealed that this is accompanied by adjustments to D_H_ identity, D_H_ reading frame, J_H_, and CDR-H3 amino acid content. These adjustments in the periphery led to wild-type levels of CDR-H3 Y101 content among transitional (T1), mature recirculating, and marginal zone B cells. However, peripheral selection proved incomplete, with failure to restore Y101 levels in follicular B cells and increased production of dsDNA-binding IgM antibodies.

## Introduction

In bone marrow and fetal liver, immunoglobulin (Ig) heavy (H) chain genes are generated *de novo* through variable (V), diversity (D), and joining (J) gene segment rearrangement and non-germline encoded N addition (1). The site of VDJ juxtaposition, which is the focus for repertoire diversification, is termed H chain complementarity determining region 3 (CDR-H3). The location of CDR-H3 at the center of the antigen binding site allows it to often play a determinative role in antigen recognition and binding (2).

Although highly variable in sequence, in both human and mouse, CDR-H3 is remarkably enriched for tyrosine (3). The sequence contributed by the D_H_ lies at the center of CDR-H3, thus many CDR-H3 tyrosines are D_H_ encoded. Each D_H_ is flanked on both sides by one turn recombination signal sequences, which allows progenitor B cells access to three reading frames (RFs) by deletion and three by inversion. Each RF encodes a distinctly different amino acid signature (4). Among these, RF1, which is the most frequently used, is enriched for tyrosine, as are J_H_1, 2, and 4 (5, 6). To test the role of D_H_ RFs in tyrosine enrichment, we created mice with altered D_H_ to promote use of non-tyrosine-enriched RF sequence. As predicted, tyrosine usage declined (7), supporting the view that natural selection of D_H_ sequence partly predetermines the amino acids at the center of the antigen binding site.

Following creation of their H chains, developing B cells must pass through a sequential series of quality control (QC) checkpoints (8) that act to test the integrity, function, or binding properties of their Ig (5). Each of these Ig properties is heavily influenced by the sequence and structure of CDR-H3. Although tyrosine (Y) enriched D_H_ RF1 is preferred in CDR-H3, threonine (T) enriched RF2, and arginine (R) and tryptophan (W) encoding RF3, important for protein-protein interaction are also used but at low levels. In addition, N addition permits the inclusion of random sets of non-germline encoded amino acids. We postulated that if CDR-H3 tyrosine were of importance to the function of the H chain, QC checkpoints might also be enlisted to enrich for tyrosine in order to balance the effect of using alternative RFs or N addition.

The first H chain QC checkpoint requires formation of a functional pre-B cell receptor (preBCR). Each μH chain (μHC) is tested for its ability to associate with the surrogate light chain (SLC) proteins VpreB and λ5 to create a functioning preBCR (9). In support of our hypothesis, we observed that H chains that successfully passed through this QC checkpoint were more likely to encode tyrosine at CDR-H3 position 101 (Y101) than those that did not.

Examination of crystallized IgG antibody–antigen complex structures revealed Y101 was often in direct contact with the bound antigen. This was not unexpected, since studies of protein ligand–receptor interfaces have shown that tyrosine is one of the three amino acids that typically make the greatest contribution to receptor binding affinity (8). Although tryptophan, the second common component of protein–protein interactions, is found in D_H_ RF3 and in J_H_1 and 3, it is only slightly more common in CDR-H3 than what would be expected by random chance alone. And there is no enrichment for arginine, the third common component (3). Together, these findings led us to the conclusion that tyrosine plays a key role in antigen binding that is so important to the developing antibody repertoire that, unlike tryptophan and arginine, both natural selection and preBCR somatic selection are recruited to the effort of favoring tyrosine, especially at Y101 (5).

In this work, we sought to test whether selection in the periphery would also promote Y101. We now report an analysis of the developing repertoire in BALB/c B lineage cells deficient in λ5 (λ5KO) and thus denied central preBCR selection. In the absence of preBCR, we confirm that selection for CDR-H3 Y101 is relaxed among immature B cells. However, with maturation in the periphery, the prevalence of Y101 increases. In particular, the sequences we obtained from transitional (T1), mature recirculating bone marrow, and marginal zone (MZ) B cells demonstrate no statistically significant differences between the prevalence of Y101 in λ5KO versus wild-type (WT) BALB/c mice. These findings suggest that in addition to the preBCR checkpoint, one or more peripheral QC checkpoints between the immature cell stage and mature, IgM/IgD bearing splenic B cell stages also favor the survival of B cells bearing Igs with CDR-H3 Y101.

## Materials and Methods

### Study Design

We sought to determine the consequences of absence of the preBCR QC checkpoint on peripheral B cell repertoire development by comparing the repertoire in late bone marrow and splenic B cell subsets isolated from BALB/c WT and λ5KO mice (6). We focused on CDR-H3, which plays a critical role in antigen binding and recognition. The CDR-H3 sequences from the WT mice have been previously published (3, 10). For λ5KO, data were obtained from six mice. All the sequences obtained from the mice are presented. All of the H chain sequences obtained from sorted bone marrow and spleen B cells were unique. Both the previously reported CDR-H3 sequences from WT mice and the new CDR-H3 sequences from the λ5KO mice are provided in Table S1 in Supplementary Material.

### Mice

All experiments and animal procedures were performed using protocols approved by the University of Alabama Institutional Animal Care and Use Committee (IACUC). BALB/c mice were bred under specific pathogen-free conditions in the UAB vivarium. λ5KO mice, originally on a C57BL/6 background (11), were backcrossed for 22 generations onto BALB/c (6).

### Flow Cytometry and Cell Sorting

Single-cell suspensions were prepared from the femurs and spleen of 8- to 12-week-old mice as previously described (4, 10). The scheme of Hardy (12) was used to identify immature and mature B cell subsets in the BM. The scheme by Allman et al. (9) was used to identify splenic transitional T1, T2 and splenic follicular B cells. The scheme by Loder et al. (13) was used to identify MZ B cells. A BD FACS Aria II instrument (BD Biosciences, Becton, NJ, USA) was used for sorting. Cells were washed, stained, and analyzed using the following labeled antibodies: anti-B220 (PB), anti-CD19 (APC-Cy7), anti-CD43, anti-BP-1 (PE) (APC), anti-B220 (APC), anti-IgM (APC), anti-CD23 (PE) and anti-CD21 (APC) [BD Pharmingen, San Diego, CA, USA], and anti-AA4.1 (PE-Cy7) (eBioscience, San Diego, CA, USA). Propidium iodide (PI) was used to identify dead cells. The sorting schemes are illustrated in Figure S1A in Supplementary Material.

### RNA Preparation, RT-PCR, and Sequencing

Total RNA isolation, VH7183 specific VDJCμ RT-PCR amplification, cloning, sequencing, and analysis were performed as previously described (10). Sequences were obtained from at least six different mice.

### Evaluation of the Extent of Apoptosis

The prevalence of apoptosis among Hardy Fractions E and F in the bone marrow, and T1, T2, follicular (FO), and MZ B cells in the spleen was determined based on PI and Annexin V staining. The FITC Annexin V Apoptosis Detection Kit II [BD Biosciences, San Jose, CA, USA] was used to stain the cells.

### Bromodeoxyuridine (BrdU) Labeling

Bromodeoxyuridine incorporation was used to assess the prevalence of B cells that had undergone cell division within the previous week. BrdU was administered in the drinking water (2%) for 1 week. Mice were then sacrificed, bone marrow and splenic cells were washed and fixed, and BrdU-positive B cells were detected using antibodies specific for BrdU using the BD BrdU-flow kit [BD Biosciences] according to the manufacturer’s protocol.

### Assessment of IgM dsDNA-Binding Antibody Titers

Titers of dsDNA-binding IgM were obtained by ELISA, as previously described (14). Plates (96-well Costar 9018, Corning Incorporated) were treated with poly-l-lysine solution for 2h and then coated with DNA sodium salt from calf thymus (D3664-2MG, Sigma-Aldrich, Saint Louis, MO, USA). Serum samples (three 1:2 serial dilutions), and HRP-labeled secondary antibodies were diluted in 1.5% BSA-PBS. Secondary antibodies against mouse IgM (1020-05) and IgG (1031-05) were obtained from Southern Biotech (Birmingham, AL, USA). To develop the ELISA, we used 100 µL of 1× TMB ELISA substrate solution (eBioscience 00-4201-56) per well and incubated the plates for 10 min in the dark. The reaction was stopped using 50 µL of 2 N H_2_SO_4_, read and analyzed with a FLUOstar Omega microplate reader (BMG Labtech, Cary, NC, USA).

### Statistical Analysis

Differences between populations were assessed where appropriate by the two-tailed Fisher’s exact test, two tailed Student’s *t*-test, or Levene’s test for the homogeneity of variance. Analysis was performed with JMP version 11 (SAS Institute, Inc., Cary, NC, USA).

## Results

In λ5KO mice, a small number of preB cells will rearrange a light chain, as well as an Igμ H chain, and thus express a conventional BCR that will enable them to advance to the immature B cell [Hardy Fraction E (12)] stage of development. At this stage, receptor editing removes many autoreactive BCRs (15, 16). As immature B cells leave the bone marrow and migrate to the spleen *via* the bloodstream, they pass through transitional T1 (17) and T2 stages (18, 19) into the mature, recirculating B cell compartment [Hardy Fraction F (12)]. T2 cells can then give rise to splenic follicular (FO) or MZ B cells (20). A fraction of MZ cells can also develop directly from the T1 population (21). FO B cells typically depend on T cells to help them produce antibodies, whereas MZ B cells are typically T cell independent.

In accordance with previous reports (11, 22), at 8–12 weeks of age mice deficient for λ5 suffered a 10-fold decrease (*p* < 0.01) in the absolute numbers of immature, Fraction E cells in the bone marrow, and in the numbers of T1 (*p* < 0.01), T2 (*p* < 0.0001), and FO (*p* < 0.0001) cells in the spleen when compared to WT (Figure S1B in Supplementary Material). Indeed, the numbers of mature, Fraction F, recirculating B cells in the bone marrow were halved (*p* < 0.01). In contrast, the numbers of MZ B cells in the spleen were statistically indistinguishable from WT.

To test whether somatic selection in the absence of preBCR would influence CDR-H3 repertoire development, we used RT-PCR and standard cloning and sequencing techniques to analyze V_H_7183-D-J-Cμ transcripts from sorted B cell subsets from λ5KO BALB/c mice. In total, we deconstructed 563 unique transcripts. This included 81 transcripts from fraction E, 90 from T1, 96 from T2, 77 from fraction F, 124 from FO, and 95 from MZ B cells. We compared these transcript sequences to 928 transcripts previously obtained from WT BALB/c mice (10, 23). This included 255 transcripts from fraction E, 102 from T1, 96 from T2, 254 from fraction F, 111 from FO, and 110 transcripts from MZ B cells (Table 1).

### Increased Use of D_H_ RF2 Coding for Non-Y Amino Acids in Early B Cell Development

**Figure 1:**
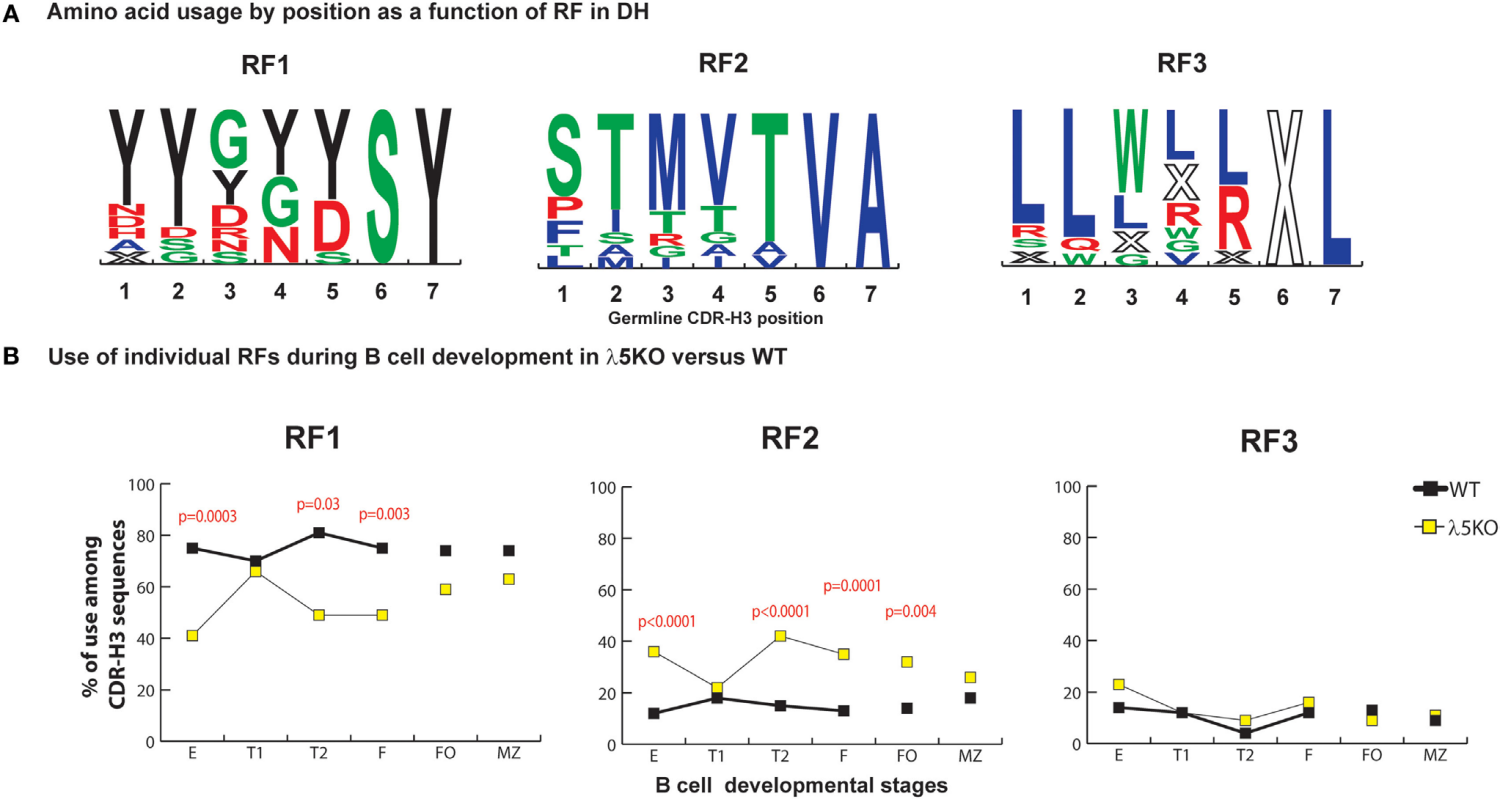
Differences in the use of D_H_ reading frames (RFs) in λ5KO versus wild-type (WT). **(A)** Amino acids in rank order according to their expression (position by position) from total germline D_H_ as a function of RF utilization. Charged amino acids are colored red, hydrophilic amino acids are colored green, hydrophobic amino acids are colored blue, and tyrosine is colored black. The size of the single letter is proportional to its prevalence. **(B)** The difference in RF usage through successive B cell developmental stages. Immature fraction (E) and mature recirculating fraction (F) cells were identified using Hardy’s scheme (12). Transitional (T1), (T2), and follicular (FO) B cells were identified using Allman’s scheme (9). Marginal zone (MZ) B cells were identified using Loder’s scheme (13). The *p*-values for differences that achieved statistical significance (*p* ≤ 0.05) are shown. CDR-H3 sequences were unique and obtained from at least six mice per group.

In WT BALB/c mice, late preB cells favor the use of RF1 in the 12 D_H_ gene segments belonging to the DFL, DSP, and DST D_H_ families (RF1:RF2 ratio of 7:1 in late preB cells, Hardy Fraction D). Use of D_H_ RF1 favors the inclusion of tyrosine in CDR-H3, while use of RF2 favors use of threonine and RF3 favors use of leucine, tryptophan, or arginine (Figure 1A). Stop codons limit RF3 expression.

In accordance with previous reports (11, 22), the deficiency of λ5 had a major effect on the relative prevalence of RF1 and RF2. When compared to WT, we observed a major reduction in the use of RF1 in fraction E (41 versus 75%, *p* = 0.0003), T2 (49 versus 81%, *p* = 0.03), and fraction F (49 versus 75%, *p* = 0.003), with a complementary increase in the use of RF2 (Figure 1B). Deficiency of λ5 appeared to have no effect on the use of RF3 in the immature B cell fraction. Selection against an H chain containing stop codons in CDR-H3 should occur equivalently at the preBCR checkpoint in WT preB cells and in immature B cells in λ5KO, since both require a signal-competent B cell receptor. However, on a relative level since there were more RF2-containing sequences, one would have expected a decrease in the relative use of RF3, unless CDR-H3s with RF3 were also being selected against by preBCR QC.

### Reduced Use of D_H_ RF2 in Transitional T1 and Mature Splenic B Cells

Although use of RF2 was more common among bone marrow immature B cells, in the periphery use of RF1 and RF2 approached that seen in WT FO and MZ B cell fractions. Use of RF2 remained more common among λ5KO FO cells than among WT FO cells (*p* = 0.004), but did not achieve statistical significance among the MZ B cell sequences. Complete convergence of RF usage between WT and λ5KO was observed among the T1 cell fraction (Figure 1B). This pattern of convergence toward the WT RF standard among the FO and MZ or T1 subsets proved to be a recurring theme.

### In the Absence of preBCR Selection, Use of D_H_ DSP Gene Segments Is Increased

Of the six mechanisms known to influence D_H_ reading frame usage and CDR-H3 amino acid content (6, 24–26), two occur at the time of VDJ rearrangement, three are associated with the preBCR checkpoint, and one is influenced by the antigen binding features of the final Ig. The first of the VDJ rearrangement mechanisms is the overwhelming preference of rearrangement by deletion over rearrangement by inversion in the H chain locus. The second is a preference for rearrangement at the sites of microhomology between rearranging gene segments. Both of these mechanisms favor rearrangement into RF1.

The first of the three mechanisms associated with the preBCR checkpoint takes advantage of the presence of an *ATG* start site upstream of D_H_ RF2 that permits production of a DJ expressed protein termed Dμ. Many Dμ proteins can associate with SLC to create a truncated preBCR, and the signals emanating from this shortened receptor promote allelic exclusion, inhibiting subsequent V→DJ rearrangement. In the absence of a complete H chain, most of these cells which will die by apoptosis. The second is dependent on the interaction between CDR-H3 101 and the SLC. The presence of a tyrosine at position 101 favors passage through the preBCR checkpoint. Most of the tyrosines at position 101 are contributed by D_H_ RF1. The third mechanism elicits the aid of stop codons of D_H_ in RF3.

**Figure 2:**
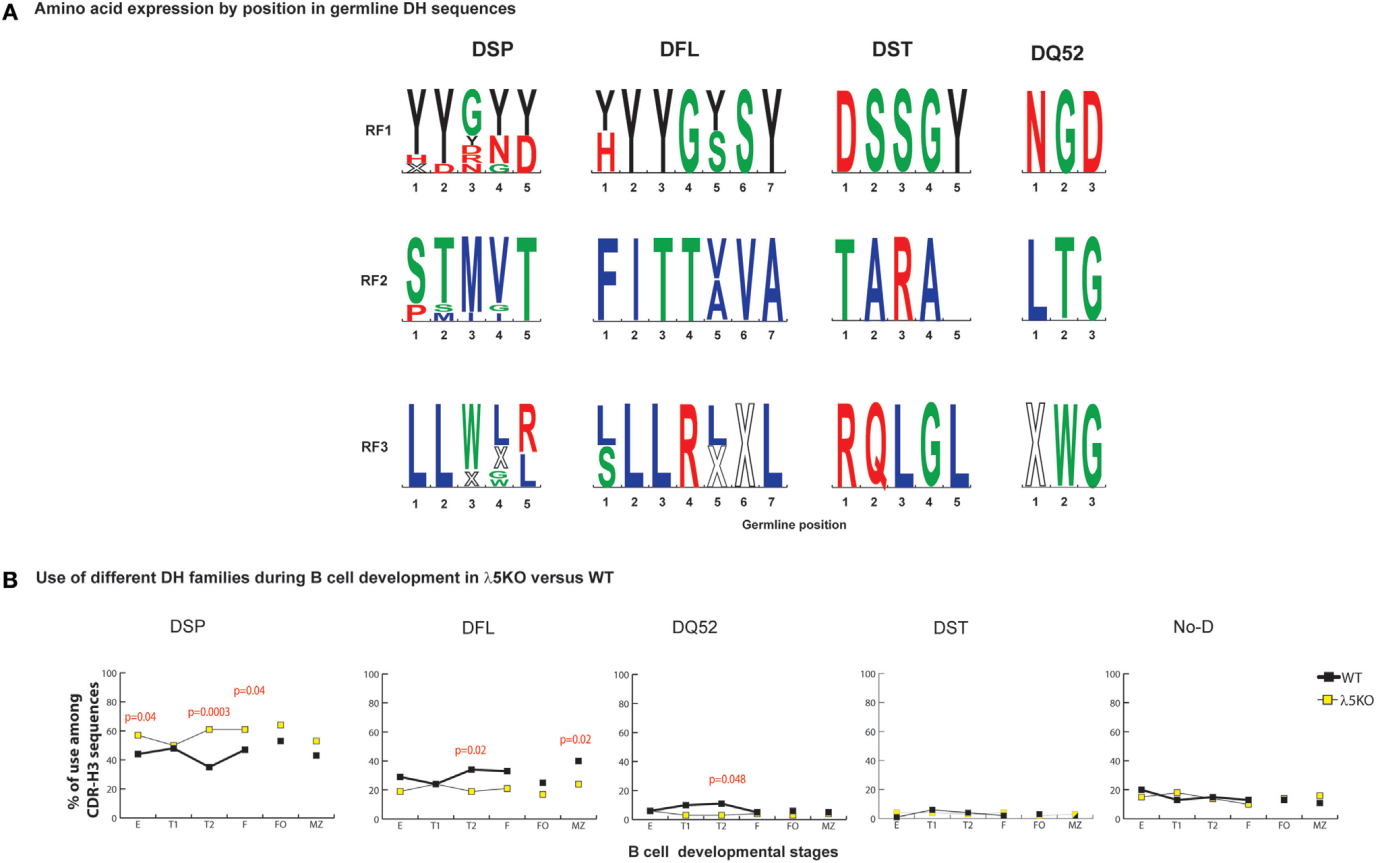
Differences in the use of D_H_ families in λ5KO versus wild-type (WT). **(A)** The rank order expression of amino acids of the different D_H_ germline sequences as a function of reading frames (RF) based on position. The top panel presents the amino acid sequences encoded by RF1, the middle panel RF2, and the lower panel RF3. The four functional D_H_ (DSP, DFL, DST, and DQ52) families are shown. Amino acids are color coded as described above. Stop codons are represented by an X. The size of the single letter is proportional to its prevalence. **(B)** Difference in use of individual D_H_ gene families at selected B cell developmental stages in λ5KO compared to WT. The *p*-values for differences that achieved statistical significance (*p* ≤ 0.05) are shown.

DQ52, the outlier D_H_ gene segment, lacks both an upstream *ATG* start site and an encoded tyrosine (Figure 2A). DQ52 RF3 contains a termination codon, but it is located at the 5′ terminus and is easily deleted by imprecise VDJ joining. Unlike the other D_H_s, DQ52 demonstrates more random RF usage.

For those cells that successfully navigate through the preBCR checkpoint, CDR-H3 sequences show disparity in the selection against use of RF2 when examined by D_H_ family. Among WT sequences, the ratio of RF1 to RF2 in WT CDR-H3 sequences using DFL or DSP D_H_ gene segments was 3:1 versus 21:1 (*p* = 0.0001), respectively. A similar 3:1 ratio of RF1 to RF2 was observed in mice limited to the use of DFL16.1 only (27), suggesting that this ratio is an inherent property of the DFL gene segment. It is possible that Dμ proteins that use DSP RF2 are more likely to form a signal-competent Dμ preBCR than those that use DFL gene segments, perhaps due to a shorter length. If negative selection against RF2 was stronger in sequences using DSP gene segment, we reasoned that absence of preBCR selection in λ5KO deficiency would be marked by increased use these gene segments when compared to WT, and this proved to be the case. DSP gene segments were used 50% more frequently among Fraction E cells from λ5KO mice than from WT Fraction E (*p* = 0.04), with a compensatory decrease in the use of DFL gene segments (Figure 2B).

To further assess the role of the D_H_ family, we analyzed sequences using DFL or DSP gene segments separately. In Fraction E, differences in reading frame usage failed to achieve statistical significance among sequences using DFL D_H_ gene segments (Figure S2 in Supplementary Material), whereas use of RF2 was significantly enhanced (*p* = 0.0001) among sequences using DSP D_H_ gene segments (Figure S3 in Supplementary Material). Use of DST, DQ52, and unidentifiable D_H_ gene segments in CDR-H3 proved almost indistinguishable between WT and λ5KO at the immature B cell stage (Figure 2B).

In support of our hypothesis that maturation in the periphery would be influenced by RF usage, analysis of CDR-H3s from λ5KO mice showed that the ratio of RF1 to RF2 usage among FO and MZ B cells, as well as cells in the T1 fraction, approached or converged toward WT values (Figure 1B). When we pooled sequences that used DFL gene segments, we demonstrated the same pattern of RF1 and RF2 usage in λ5KO as in WT especially in T1, FO, and MZ compartments (Figure S2 in Supplementary Material). Among pooled sequences using DSP gene segments, the difference in RF usage between λ5KO and WT diminished with development. While statistically significant differences remained in the follicular fraction, albeit with evidence of convergence toward WT when compared to immature B cells, differences between WT and λ5KO among T1 and MZ B cells failed to achieve statistical significance (Figure S3 in Supplementary Material).

### Enrichment for J_H_3 and Depletion of J_H_2

**Figure 3:**
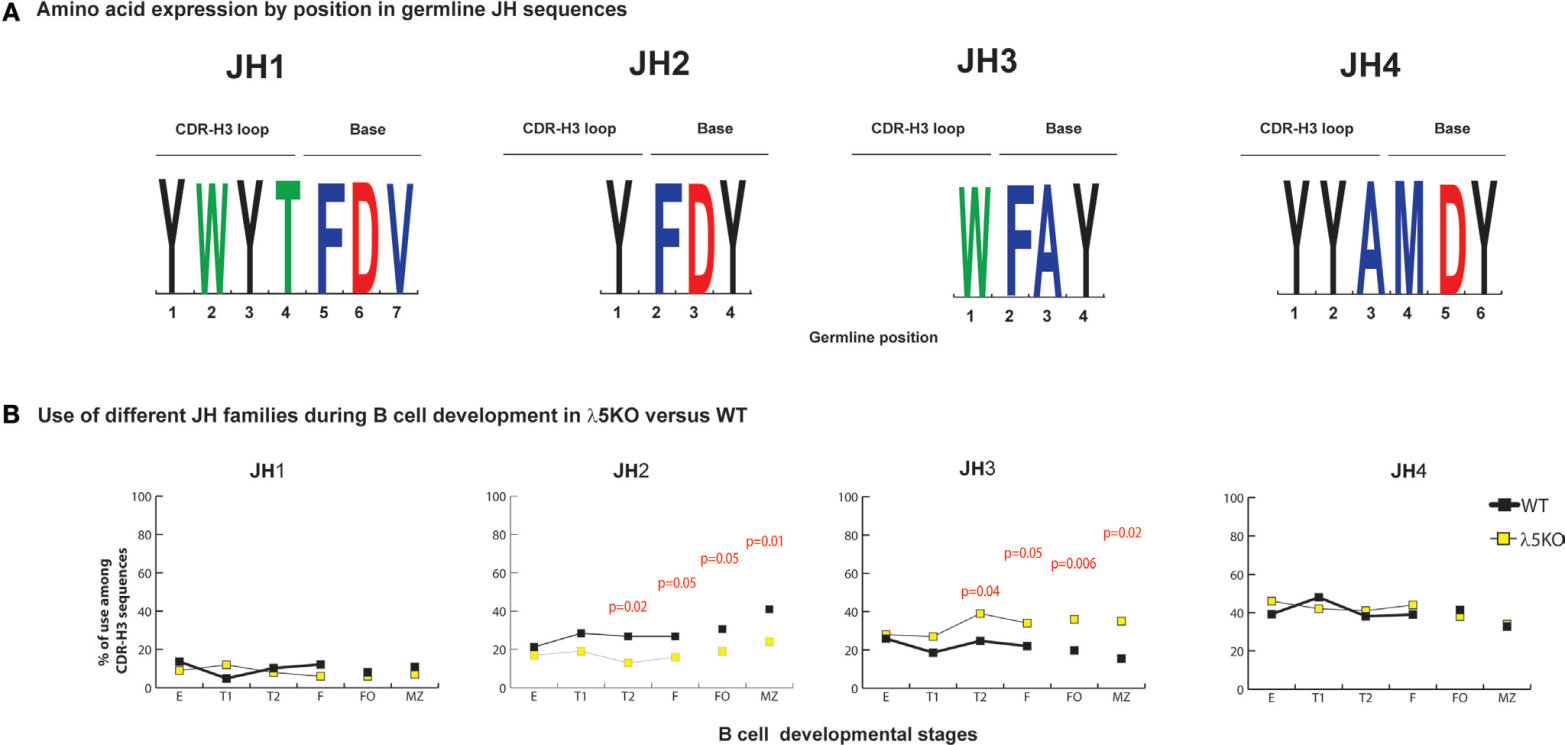
Differences in the use of J_H_ in λ5KO versus wild-type (WT). **(A)** The amino acid sequences of J_H_1, J_H_2, J_H_3, and J_H_4 that contribute to CDR-H3. The relative contributions of each J_H_ sequence to the CDR-H3 loop and base are shown. The size of the single letter is proportional to its prevalence. **(B)** Representation of the use of different J_H_ in selected bone marrow and splenic B cell subsets. The *p*-values for differences that achieved statistical significance (*p* ≤ 0.05) are shown.

On average, the J_H_ sequence contributes to ~one-fourth of the amino acids within the CDR-H3 (Figure 3A). J_H_ gene segments have only one reading frame, thus their amino acids are already predetermined. J_H_2 and J_H_4 genes contribute Y to the CDR-H3 loop, J_H_1 can contribute both Y and W, but J_H_3 contributes only W (Figure 3A).

Microhomology between the 3′ terminus of the D_H_ and the 5′ terminus of the J_H_ is the second mechanism operating at the time of VDJ rearrangement that helps regulate reading frame preference (25, 28). The extent of microhomology varies by gene segment. J_H_1 shares two to nine nucleotides with the various D_H_, J_H_2 shares two to six, J_H_3 shares zero to five, and J_H_4 shares two to three. Microhomology promotes recombination of J_H_1, 2, and 4 into DFL, DSP, and DST RF1. Microhomology between J_H_3 and the DFL and DST gene segments occurs in D_H_ RF1. However, for DSP, the two to five nucleotides of microhomology with J_H_3 occurs in the middle, rather than the 3′ terminus, of the D_H_ and it promotes recombination into RF2. Microhomology in the middle of the D_H_, rather than at the 3′ terminus reduces the effectiveness of shared sequence in influencing reading frame usage.

In immature B cell Fraction E, use of all four J_H_ among λ5KO B lineage cells was statistically indistinguishable from WT (Figure 3B; Figures S4 and S5 in Supplementary Material). When compared to WT, the relative use of J_H_1 and J_H_4 remained unchanged in the periphery among the λ5KO B cell subsets. However, divergence with maturation was noted for J_H_2 and J_H_3, with use of Y encoding J_H_2 decreasing (*p* = 0.05 and 0.01 for FO and MZ, respectively) and use of W-encoding J_H_3 increasing (*p* = 0.006 and 0.02 for FO and MZ, respectively) (Figure 3B). When examined by D_H_ family, there was enrichment for J_H_3 in the recirculating fraction F. However, among the FO and MZ B cells that used DFL gene segments, the differences in using J_H_2 in λ5KO versus WT did not achieve statistical significance (Figure S4 in Supplementary Material). In contrast, among the mature (Fraction F, FO and MZ) cells that used DSP gene sequences, the MZ cells demonstrated a significant decrease in the use of J_H_2 matched by an increase in the use of J_H_3 (*p* = 0.02) (Figure S5 in Supplementary Material). Thus, the convergence of the WT and λ5KO repertoires in peripheral B cell subsets was incomplete when it came to sequences using J_H_2 or J_H_3. Neither of these gene segments typically contributes to the amino acid at position 101, because they are too short.

### Effect of Alterations in D_H_, D_H_ RF, and J_H_ Usage on Global CDR-H3 Loop Amino Acid Content

At a global level, the loss of λ5 led to statistically significant decreases in the use of D_H_ tyrosine (RF1), serine (RF1), and glycine (RF1) in one or more of the B cell subsets studied. Conversely, λ5 deficiency led to statistically significant increases in threonine (RF2), as well as proline (created by V→D joining), alanine (RF2), and valine (RF2). Although there was no statistically significant increase in the use of RF3, the use of arginine (mostly RF3, but some RF1 and RF2) and tryptophan (RF3) also increased (Figure S6 in Supplementary Material).

**Figure 4:**
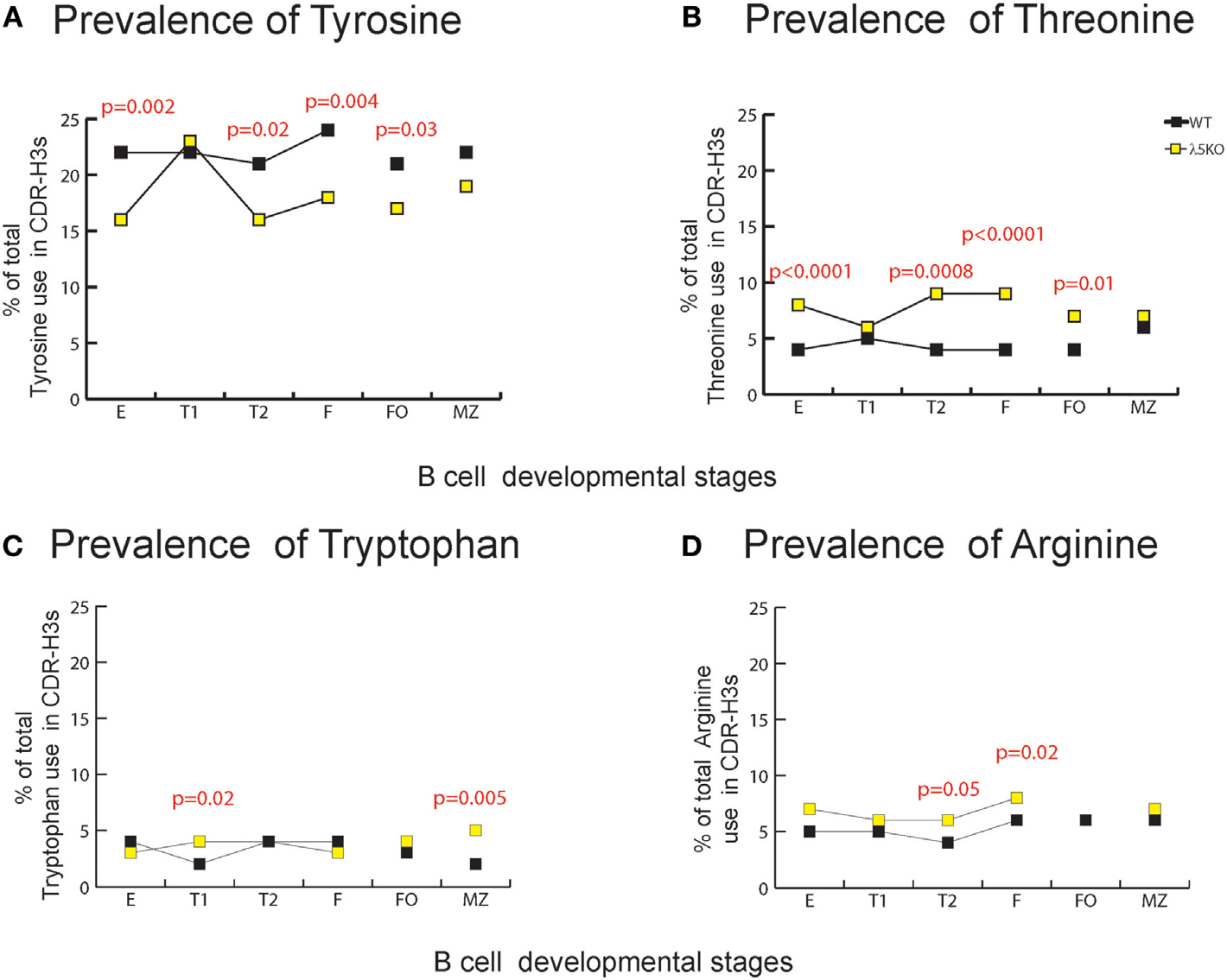
Representation of CDR-H3 amino acids important for protein–protein interaction in λ5KO versus wild-type (WT). Prevalence of **(A)** tyrosine, **(B)** threonine, **(C)** tryptophan, and **(D)** arginine amino acid expressed in CDR-H3 loop sequences obtained from λ5KO versus WT mice in selected B cell subsets. The *p*-values for differences that achieved statistical significance (*p* ≤ 0.05) are shown.

Due to their often-major contribution to binding affinity at protein ligand–receptor interfaces, we focused our attention on changes in tyrosine, tryptophan, and arginine (29). We also focused on threonine, which is characteristic of RF2 usage (Figures 1A and 2A). With the exception of T1 and MZ, loss of λ5 led to a statistically significant (*p* = 0.02 to *p* = 0.002) decrease in the prevalence of tyrosine in the CDR-H3 loop (Figure 4A). Conversely, again except for T1 and MZ, there was a statistically significant compensatory increase in the use of threonine (*p* = 0.01 to *p* < 0.0001) (Figure 4B). Loss of λ5 led to a statistically significant increase in tryptophan in T1 and MZ (*p* = 0.02 and *p* = 0.005, respectively) (Figure 4C). Finally, there was an increase in the prevalence of arginine that approached significance in T2 (*p* = 0.05) and reached significance in fraction F (*p* = 0.02) (Figure 4D).

To gain further insight into the source of the increase in tryptophan in the λ5KO mice, we examined the relative contribution of tyrosine from the amino terminus of J_H_2, and the tryptophan from the amino terminus of J_H_3. As noted above, use of J_H_3 is increased in MZ B cells in the λ5KO mice (Figure 3B). Commensurate with the even greater relative use of J_H_3 versus J_H_2 in the MZ; of the sequences that used J_H_2, 49% of the WT and 43% of the λ5KO sequences retained the tyrosine codon at the 5′ terminus of the J_H_. And, among the MZ sequences that used J_H_3, only 12% of the WT versus 61% of the λ5KO sequences retained the tryptophan codon (*p* = 0.04). Thus, the increase in tryptophan in CDR-H3 was most apparent at the carboxy terminus of the CDR-H3 loop, beyond position 101, and reflected selection for use of J_H_3 gene segments with limited nucleotide nibbling at the amino terminus of the J_H_.

### Peripheral Selection for Tyrosine at CDR-H3 Position 101

**Figure 5:**
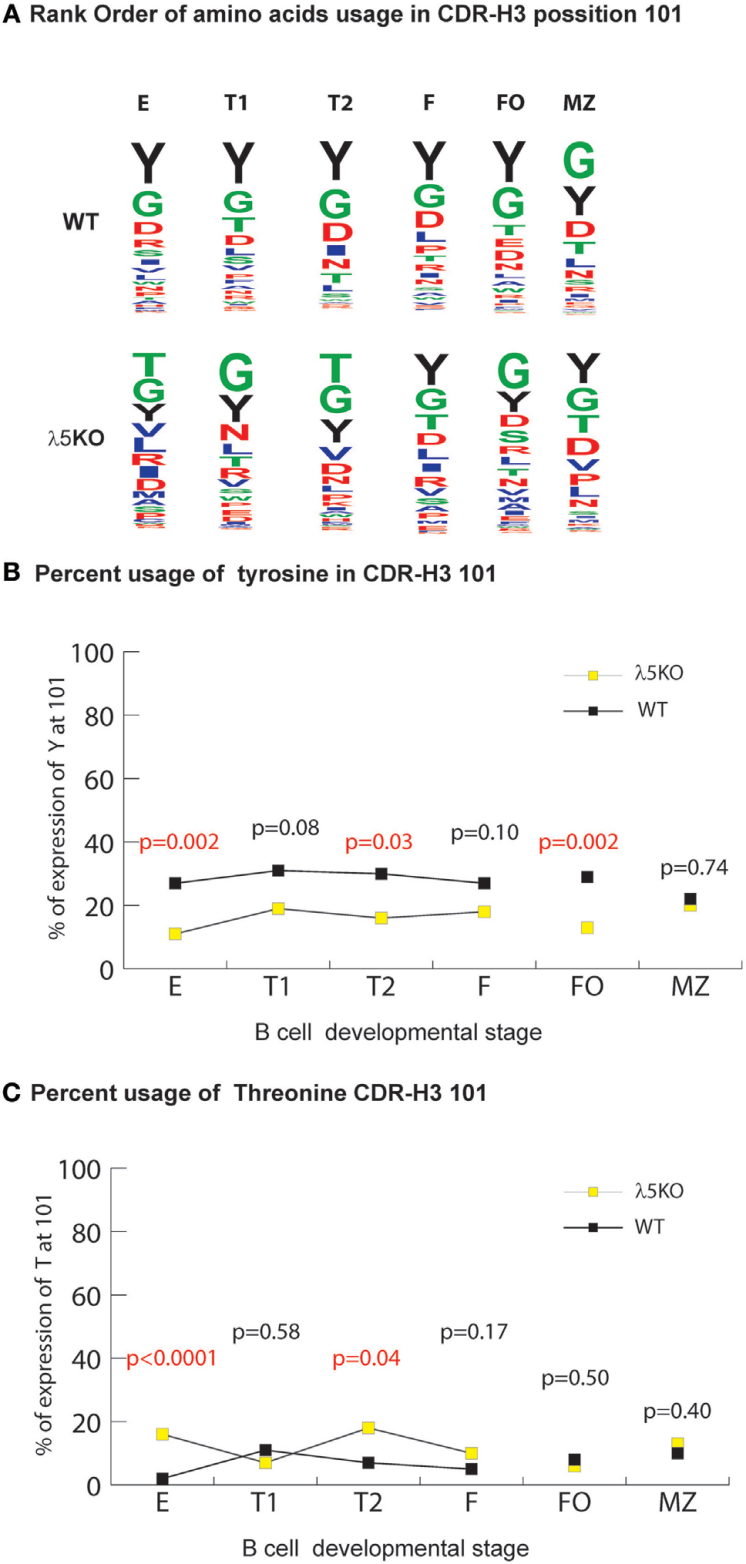
Selection for CDR-H3 Y101 in λ5KO versus wild-type (WT). **(A)** The use of different amino acids in CDR-H3 position 101 in selected B cell subsets. Upper panel lists WT CDR-H3 amino acids at position 101 in rank order; the lower panel lists the homologous amino acids in λ5KO mice, also in rank order. The size of the single letter is proportional to its prevalence. **(B)** Prevalence of Y101 and **(C)** T101 in selected B cell subsets. The *p*-values for the differences between λ5KO and WT are shown. Those *p*-values that achieved statistical significance (*p* ≤ 0.05) are marked in red.

The most prominent effect of passage through the preBCR checkpoint on CDR-H3 amino acid content occurs at position 101 where there is particular enrichment for tyrosine (6). As expected, in the absence of preBCR selection Y101 prevalence among the immature B cell fraction was halved compared to WT (*p* = 0.002) (Figure 5B). The prevalence of Y101 in the λ5KO T1, T2, and FO subsets remained depressed (*p* = 0.08, *p* = 0.03, and *p* = 0.002, respectively). This is most remarkable for RF1 encoded Y101 in the FO compartment (Figure S7 in Supplementary Material). In contrast, maturation in the periphery led to convergence in the prevalence of Y101 between λ5KO and WT in the recirculating, mature Fraction F, and MZ compartments.

For this facet of repertoire development, the differences in the relative prevalence of glycine and threonine at position 101 (G101 and T101) among the various B cell compartments were also remarkable. In both λ5KO and WT, glycine was the first or second most common amino acid in all the compartments studied (Figure 5A). Conversely, in spite RF2 being heavily enriched for threonine, there was convergence in the use of T101 in spite of the increased use of RF2 in λ5KO F and FO (Figure 1B) when compared to WT (Figure 5C). Thus, the selective pressures on the use of T101 manifested earlier in development than Y101 and included the FO, as well as the F and MZ compartments.

### Increased Apoptosis with Peripheral B Cell Development in λ5KO B Cells

**Figure 6:**
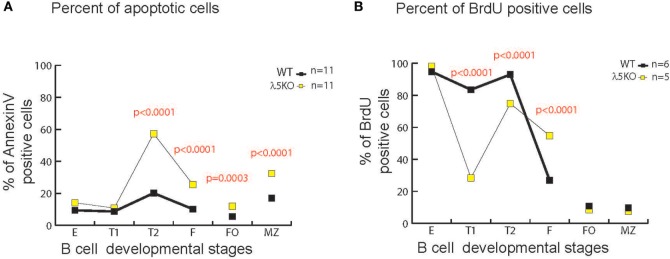
Apoptosis and cell turnover in λ5KO versus wild-type (WT). **(A)** Comparison of the percentage of Annexin V positive (apoptotic) cells in λ5KO versus WT mice. Data were obtained from 11 mice per group. **(B)** Comparison of bromodeoxyuridine (BrdU)-positive cells among selected B cell subsets as a measure of cell turnover in λ5KO versus WT mice. Student’s *t*-test was used for statistical analysis. The *p*-values for differences that achieved statistical significance (*p* ≤ 0.05) are shown.

In the bone marrow, enrichment for RF1 encoded Y101 reflects, in part, increased apoptosis of B cells containing RF2 encoded amino acids at position 101 due to preBCR selection (6). To test whether there was a correlation between increased cell death and increased use of Y101, we assessed the percentage of total Annexin V positive λ5KO cells relative to WT in the various B cell subsets. No differences in the level of apoptosis were observed in fractions E and T1 (Figure 6A). In contrast, λ5KO T2 (*p* < 0.001), recirculating mature Fraction F (*p* < 0.0001), splenic FO cells (*p* = 0.0003), and splenic MZ (*p* < 0.001) B cells all showed enhanced apoptosis. In particular, the difference between T1 and T2 is striking with 15% of T1 cells undergoing apoptosis in both WT and λ5KO, versus 10% of WT T2 and 60% of λ5KO T2 (Figure 6A).

### Decreased Turnover in λ5KO Mature B Cells

To gain further insight into the kinetics of the B cell homeostasis in the λ5-deficient mice, we used BrdU incorporation to evaluate cell turnover. As expected, after one week’s exposure to BrdU in the drinking water, virtually all of the immature fraction E cells in both WT and λ5KO had incorporated BrdU (Figure 6B), indicating that they had recently undergone cell division. This suggested that the majority of Fraction E cells were also recent products of B cell differentiation and preB cell proliferation in the bone marrow. However, unlike the mature, recirculating Fraction F cells in the WT mice, where only one quarter of the mature cells were recent emigrants (BrdU positive), slightly more than half of the λ5KO Fraction F cells mice had incorporated BrdU (*p* < 0.0001). This suggested that the life expectancy of recent emigrants from the λ5KO, many of whom lacked Y101, was lower than WT.

In marked contrast, less than 10% of the FO and MZ B cells in both λ5KO and WT had incorporated BrdU. This suggested that the vast majority of the FO and MZ B cells in both strains of mice had been resident in the body for more than 1 week, during which time they had been bathed by peripheral antigens.

Intriguingly, in λ5KO, a much greater fraction of the T1 population had failed to incorporate BrdU than in WT. Approximately 17% of the WT and 71% of the λ5KO T1 fraction was sedentary (*p* < 0.0001). Together with our previous data, this suggested that the T1 cell fraction contains a significant subset of cells that had last undergone cell division more than a week prior and thus were relatively long-lived. These resident T1 cells had undergone selection for the BCR that allowed the repertoire in the λ5KO to converge in reading frame and amino acid content to that observed in WT.

### Production of Autoreactive Anti-dsDNA Antibodies in λ5KO Mice

**Figure 7:**
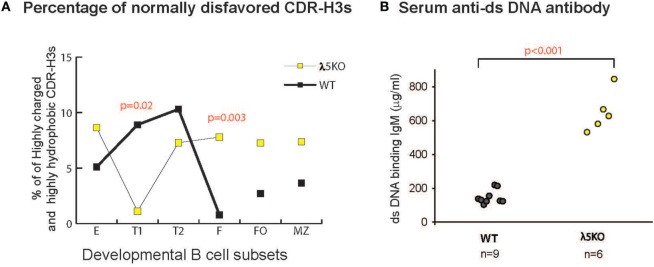
Prevalence of disfavored highly hydrophobic or highly charged CDR-H3s and dsDNA-binding IgM antibodies in λ5KO versus wild-type (WT). **(A)** Comparison of the percentage of normally disfavored highly hydrophobic or charged CDR-H3 sequences obtained from λ5KO versus WT in selected B cell subsets. A modified Kyte-Doolittle scale was used to assess hydrophobicity (30). Highly hydrophobic CDR-H3 sequences were identified as having an average hydrophobicity of greater than 0.6 on the scale, while highly charged are less than −0.7. The *p*-values for differences that achieved statistical significance (*p* ≤ 0.05) are shown. **(B)** Serum levels of dsDNA-binding IgM at 8 weeks of age in λ5KO versus WT mice. Data represent analysis of 6 and 9 mice per group, respectively. The concentration of dsDNA-binding IgM was measured in microgram per milliliter. Student’s *t*-test was used for statistical analysis.

An alternative approach to evaluate the physicochemical characteristics of the CDR-H3 repertoire is to calculate the average hydrophobicity of the amino acids in the CDR-H3 interval using the Kyte-Doolittle scale (3). During normal B cell development in WT BALB/c mice, there is a focusing of the repertoire to reduce or limit recirculating mature B cells with highly hydrophobic or highly charged CDR-H3 intervals (Figure 7A) (3). Many of these sequences contain valine (RF2) or leucine (RF3), or arginine (mostly RF3, but some RF2 and RF1). In WT mice, splenic FO and MZ B cell subsets retain some of these sequences, with the MZ in particular retaining highly charged, arginine-enriched CDR-H3s.

In the absence of preBCR selection, we observed an increase in the prevalence of highly charged and highly hydrophobic CDR-H3s among all the B cell subsets studied, except for T1 where selection against these intervals was very strong.

Strains of mice that fail to limit the prevalence of highly charged CDR-H3s, especially those enriched for arginine, demonstrate increased susceptibility to the production of double-stranded DNA-binding antibodies (26, 31, 32). To test the effect of the loss of preBCR selection on autoantibody production, we measured the titers of dsDNA-binding IgM and IgG antibodies in the serum of 8-week-old homozygous WT and λ5KO BALB/c mice by ELISA. A significant increase in dsDNA-binding IgM was observed in the λ5KO mice (*p* < 0.001) (Figure 7B), but not in IgG (data not shown). No evidence of proteinuria was obtained in these mice (data not shown).

## Discussion

Even with modest assumptions, the potential diversity of the pre-immune antibody repertoire created by V(D)J rearrangement and N addition exceeds 10^16^. Somatic hypermutation, which typically occurs after antigen exposure with T cell help, permits further exponential diversification of the antigen-experienced repertoire. The power of these processes has led to the concept of the antibody repertoire as being composed of near-infinite diversity with a high likelihood of including within it one or more antibodies with the specificity needed to provide prophylactic or therapeutic benefit to antigen challenge. However, practical thinking reveals at least three major limitations to this concept. First, at any one time the number of B cells, and thus the number of different antibodies, in the organism is limited. Second, random diversity will generate many ineffective or unneeded antibodies. Each of these failed antibodies imposes energy costs on the host organism. Third, random diversity also engenders random autoreactivity, and thus the risk of morbidity from autoimmune disease. Clinical experience reveals a fourth limitation, which is time. Failure to produce protective antibody within a 2- or 3-week period after infection can lead to morbidity and mortality in the host.

We previously postulated that pressure to minimize morbidity and mortality, and thus enhance reproductive fitness, which is the driving force in evolution, would encourage the development of mechanisms that could be used to guide and thus potentially “optimize” the development of the antibody repertoire (4). In previous studies, we showed that in spite of a potential diversity of 10^16^, less than 10^2^ sequences were needed to reveal major biases in CDR-H3 amino acid content. These were shown to result, in part, from natural selection of D_H_ sequence and reading frame preference (7). In genetically altered mice with D_H_ sequences that promote the use of alternative, i.e., non-evolutionarily preferred, amino acids, reductions in total serum Ig levels (7), changes in antigen-specific antibody responses (5, 7), changes in patterns of epitope recognition and repertoire development during affinity maturation (5, 33), and impaired protection against a variety of infectious agents (7, 34) were all observed.

Analysis of the developing repertoire in the bone marrow and in the periphery of WT mice also revealed evidence of somatic selection of CDR-H3 amino acid usage. In particular, we noted a stair step increase in the use of tyrosine and a reduction in the use of highly charged and highly hydrophobic CDR-H3s in both the transition from the early to late pre-B cell stages, coincident with central preBCR selection (7), and in the transition from the immature to mature, recirculating B cells, splenic cells, and cells in the peritoneal cavity (7, 10). We postulated that these somatic processes might be needed to help control repertoire diversity engendered by N addition and exonuclease nibbling, which could not be constrained by natural selection alone. Remarkably, the extent of selection varied between strains of mice. In particular, strains prone to retain highly charged CDR-H3s in their mature, recirculating B cell pool are coincidentally more susceptible to production of DNA-binding antibodies (31, 32). These findings suggested that processes of somatic selection of the repertoire both centrally and peripherally were being superimposed on the constraints on the repertoire imposed by natural selection of Ig gene sequence and that the relative strength of these selective processes could vary and thus influence patterns of antibody reactivity.

To begin to identify the forces acting centrally to select the CDR-H3 repertoire, we evaluated CDR-H3 before and after passage through the pre-B cell receptor (preBCR) QC checkpoint (6). Not only did we confirm selection for tyrosine in CDR-H3, we observed that selection for tyrosine was most intense at the top of the CDR-H3 loop, i^.^e., position 101, where it could interact with an amino acid motif (RDR) in the VpreB component of the preBCR that was conserved between human and mouse.

The major role of Ig, either as a soluble antibody or as an antigen receptor on the surface of a B cell, is to bind a ligand. It thus seems reasonable to postulate that Igs are subject to the same rules of receptor–ligand interaction efficiency as monospecific receptor–ligand pairs. Our attention was thus drawn to the observation that tyrosine is one of three amino acids that typically make the greatest contribution to binding affinity at many protein ligand–receptor interfaces (29). The use of selective forces to enrich for tyrosine in the developing repertoire, prior to antigen exposure, could be explained if the antibody repertoire had been selected by evolution to promote its role as an effective and efficient binder of ligands, which we hypothesized would benefit from the presence of tyrosine. Thus, SLC could not only act as a stand-in for conventional light chains to test for the ability of a heavy chain to pair with a light chain, but also as a stand-in for a whole range of potential antigens that would be more effectively neutralized by antigen binding sites that contain tyrosine to optimize the bond between antibody and antigen (6).

If enrichment for tyrosine in CDR-H3 by evolutionary and preBCR selection helps optimize the antibody repertoire for antigen binding, we postulated that the prevalence of tyrosine would increase as B cells become increasingly dependent on beneficial interactions with antigen to survive. To test this hypothesis, we focused on the prevalence of tyrosine as a function of maturation among the CDR-H3 sequences expressed by peripheral B cells that had not been subjected to preBCR QC selection. In the absence of the SLC, only a few preB cells manage to progress to the immature B cell stage. Mechanisms explaining leakiness of the preBCR checkpoint in SLC-deficient mice include premature rearrangement of light chain genes before heavy chains (35), use of certain μHC that can signal independently of the preBCR (36, 37), or binding to a surrogate κ LC protein (38, 39). Leaky cells that bypass preBCR selection can engage the mechanism of allelic exclusion and undergo proliferation. Cells that prematurely rearrange the L chain gene can pass directly to the immature B cell stage or undergo light chain receptor editing. Cells that express an μHC capable of signaling without L chain and cells that express a surrogate κ LC protein can rearrange an L chain gene after proliferation. All of these leaky cells can thus ultimately express a conventional BCR (Igμ plus a κ or λ LC) and enter the immature B cell compartment, where they are phenotypically indistinguishable from B cells that have successfully navigated the preBCR checkpoint. At this stage, another checkpoint, receptor editing, will attempt to replace autoreactive BCRs (15, 16). Many immature B cells do not undergo receptor editing because their Igs were not autoreactive to begin with.

Immature B cells that pass the receptor editing stage leave the bone marrow and migrate to the spleen where they develop into early transitional T1 cells (17), which then develop into late transitional T2 cells (18, 19). Late transitional T2 cells give rise to either follicular or MZ B cells (20). A fraction of the T1 population develops directly into MZ cells (21). The follicular compartment is the site for T-dependent antibody responses, whereas B cells in the MZ do not necessarily need T cell help.

In the λ5KO mice that lack preBCR selection, we observed major changes in the immature B cell repertoire in the portion of the CDR-H3 contributed by D_H_, whereas global V_H_ and J_H_ usage patterns were mostly, although not completely, unaffected. The changes contributed by the D_H_ were D_H_ gene family specific. Sequences containing DSP D_H_ gene segments were most affected, largely due to changes in the RF1/RF2 ratio, followed by DFL family D_H_. DST and DQ52 containing sequences were minimally affected, although the number of DST sequences was small. The reason for the disparity between DSP and DFL sequences is unknown, but it is possible that the sequence of DSP-containing CDR-H3s yields more stable Dμ -containing, truncated preBCRs than those that contain DFL sequence and thus are more effective at initiating the process of allelic exclusion, shutting down V→DJ rearrangement and thus condemning the preB cell to an apoptotic cell death.

The effect of the change in RF preference on λ5KO CDR-H3 amino acid content was most marked for tyrosine and threonine. Use of tyrosine, mostly from RF1, declined both in CDR-H3 as a whole and at position 101 in specific, with a compensatory increase in the use of threonine from RF2. Tryptophan and arginine were minimally affected. These changes in the repertoire were associated with changes in cell turnover among recent bone marrow emigrants. Changes in the percent of cells that had incorporated BrdU over seven days exposure revealed that approximately 55% of the λ5KO, but only 27% of WT recirculating mature B cells (*p* < 0.0001), were recent bone marrow emigrants, confirming increased turnover. The percent of immature and T1 B cells undergoing apoptosis was indistinguishable from WT. However, a three-fold increase in cell death was observed in T2 transitional cells with a lesser, but still statistically significant, increase among recirculating mature B cells and both follicular and MZ B cells in the spleen. This suggested that the loss of the preBCR checkpoint had led to the production of a large number of B cells that were ill-equipped by their antigen receptors to survive in the periphery.

In λ5KO spleen, turnover among the follicular and MZ B cells was greatly diminished. The T1 subset also appeared to contain greater numbers of B cells that had not undergone recent cell division and were thus resting. It is possible that there are two populations of cells within the T1 gate in the λ5KO mice. The first population, which has incorporated BrdU, represents recent emigrants from the bone marrow that are progressing through normal transitional development to the T2 stage. The second population, which bears the same surface markers, is a B cell subset with a longer lifespan. It is possible that this population may belong to the MZ lineage, or it may be an entirely novel population. Although this resting population is closer to the MZ in terms of the prevalence of Y101, the pattern of reading frame usage, and the pattern of DH usage, the pattern of JH usage is quite different. Thus, these T1 cells offer a repertoire that is distinct from mature MZ cells, and well as FO cells. A dip in the percentage of cells that have incorporated BrdU is also seen among the WT T1 cell subset, although it is much less prominent than in the λ5KO (Figure 6B). This raises the possibility that both WT and λ5KO mice contain a resting population of cells within the T1 gate. Their increased prevalence in λ5KO may reflect the lower percentage of recent bone marrow emigrants that are viable in the periphery. Evidence of BCR selection supports the view that this more long-lived population has survived one or more BCR-mediated checkpoint steps in the periphery. The precise derivation of this potentially novel T1 subset is a subject of ongoing study in our laboratory.

In the periphery, a longer lifespan in individual B cell compartments appears to correlate with enrichment for tyrosine in general and in specific. This especially notable in the MZ where the prevalence of Y101 was indistinguishable between λ5KO and WT. Thus, peripheral selection of the repertoire appears to attempt to recreate what we would postulate to be an optimal CDR-H3 repertoire, enriched for Y101. Since a tonic signal through the BCR promotes B cell survival in the periphery (40), we postulate that convergence of the two repertoires is most evident in the MZ fraction because these cells are enriched for natural antibodies that recognize self and altered self-antigens and thus are more likely to receive tonic stimulation. In the absence of this tonic signal, selection for Y101 in specific appears less evident in the follicular fraction, permitting this subset, which is more likely to engage T dependent antigens, greater diversity.

Although we observed convergence of the λ5KO and WT repertoires, the ability of peripheral selection to recreate an optimal, WT-like repertoire appears incomplete. This is evident in the increase in the prevalence of tryptophan at the terminus of the CDR-H3 loop in λ5KO caused by increased use of J_H_3, in the increased use of threonine associated with increased use of RF2, and in the increased use of arginine. The sixth and final mechanism used to bias reading frame usage is the elimination of highly hydrophobic and highly charged CDR-H3 sequences, most of which are derived from RF2 or RF3. Many of the highly charged CDR-H3 sequences contain arginine, which is associated with a higher likelihood of being a part of DNA binding antibodies (26, 41). The preBCR checkpoint appears essential to control B cells that express these types of antibodies. In the absence of the preBCR, receptor editing may not be sufficient to adequately remove them. This conclusion is supported by the observation that there was no increase in the level of apoptosis in the immature B cell pool.

Our findings support the view that in addition to central preBCR selection, there is somatic selection at the transitional T1 and MZ stages that favors the survival of B cells bearing Igs with hydrophilic CDR-H3s containing Y101. This second checkpoint selection does not appear to have the power to completely normalize the repertoire, and thus allows B cells with suboptimal, e.g., self-reactive, specificities to survive beyond the T1 stage. Cells bearing these normally disfavored antibodies can then produce antibodies with potentially pathogenic specificities, such as dsDNA binding, or ineffective antibodies leading to increased cell death and cell turnover.

We conclude from this work that in addition to natural selection of D_H_ sequence and specific somatic selection for Y101 by the preBCR checkpoint, enrichment for Y101 in the pre-immune repertoire is also the consequence of peripheral selection. This could reflect selection by exposure to self and altered self-antigen due to the higher likelihood that an Ig containing Y101 will be more likely to be an effective binder of antigen. This could be more commonly true for MZ cells that are less T cell dependent than FO cells, and perhaps either more dependent or more likely to be exposed to self-antigens that prefer RF1 encoded CDR-H3s.

Both in the WT repertoire and the λ5KO repertoire, there are many CDR-H3 sequences that do not contain Y101, and Y101 is not a requisite for an antibody to be able to bind antigen. However, our findings suggest that a primary outcome of natural and somatic selection of the preimmunize repertoire is enrichment for Y101. These findings support the view that there can be features of the antigen binding site that promote optimized antigen binding, and thus serve to enhance the efficiency of an antibody generating process that is likely to produce many irrelevant or even hazardous antigen binding sites. In this sense, Ig can be viewed as a classic receptor, following the same rules as more monospecific and invariant receptors on cells that also populate the body.

## Ethics Statement

All experiments and animal procedures were performed using protocols approved by the University of Alabama Institutional Animal Care and Use Committee (IACUC).

## Author Contributions

MK designed, performed the experiments, performed the statistical analysis, and prepared the first draft of the manuscript. TB assisted in obtaining the CDR-H3 sequences. AE helped in statistical analysis. PB provided scientific insights in preBCR biology. HS directed the work and edited the paper.

## Conflict of Interest Statement

The authors declare that the research was conducted in the absence of any commercial or financial relationships that could be construed as a potential conflict of interest.

## Supplementary Material

Figure S1 Characterization and isolation of peripheral B cell subsets in λ5-deficient (λ5KO) and wild-type (WT) mice. **(A)** Flow cytometry profile used to isolate selected B cell subsets. Bone marrow immature B cells (Hardy Fraction E) and mature recirculating B cells (Hardy Fraction F) were isolated according to the scheme by Hardy and Hayakawa (12). Transitional T1, T2, and follicular (FO) cells were isolated according to the scheme by Allman et al. (9). Marginal zone (MZ) B cells were isolated according to the scheme by Loder et al. (13). At least six mice from λ5-deficient (λ5KO) mice were used to obtain cells for RNA extraction and sequencing. **(B)** Comparison of the absolute B cell numbers of immature and mature peripheral B cell subsets as a function of B cell development in λ5KO versus WT. Data were obtained from five mice per group. Student’s *t-*test was used for statistical analysis. Significance is illustrated as **p* < 0.05, ***p* < 0.01, ****p* < 0.001, and *****p* < 0.0001.

Figure S2 Differences in the use of D_H_ DFL family reading frames (RFs) in CDR-H3s from λ5KO versus wild-type (WT). The percentage of RF usage in DFL containing CDR-H3 sequences obtained from λ5KO versus WT in the studied B cell subsets. The *p*-values for differences that achieved statistical significance (*p* ≤ 0.05) are shown.

Figure S3 Differences in the use of D_H_ DSP family reading frames (RFs) in CDR-H3s from λ5KO versus wild-type (WT). The percentage of RF usage in DSP-containing CDR-H3 sequences obtained from λ5KO versus WT in the studied B cell subset. The *p*-values for differences that achieved statistical significance (*p* ≤ 0.05) are shown.

Figure S4 Differences in the use of J_H_ in DFL family containing CDR-H3s from λ5KO versus wild-type (WT). The percentage of sequences from λ5KO versus WT using one of the four J_H_ gene segments among CDR-H3s containing D_H_ DFL gene segments among selected B cell subsets. The *p*-values for differences that achieved statistical significance (*p* ≤ 0.05) are shown.

Figure S5 Differences in the use of J_H_ in DSP family containing CDR-H3s from λ5KO versus wild-type (WT). The percentage of sequences from λ5KO versus WT using one of the four J_H_ gene segments among CDR-H3s containing D_H_ DSP gene segments from selected B cell subsets. The *p*-values for differences that achieved statistical significance (*p* ≤ 0.05) are shown.

Figure S6 Distribution of individual amino acid usage in the CDR-H3s cloned from selected B cell subsets from λ5KO versus wild-type (WT). Upper panel, the percentage use of each individual amino acid, arranged in order of hydrophobicity, in the CDR-H3 loops of sequences cloned from selected λ5KO B cell subsets. Middle panel, the difference in percentage use of each amino acid in the CDR-H3 loops of λ5KO minus WT. Bottom panel, the percentage use of each individual amino acid, arranged in order of hydrophobicity, in the CDR-H3 loops of sequences cloned from selected WT B cell subsets. Significance values are listed as “*”, *p* ≤ 0.05; “**”, *p* ≤ 0.01; “***” and *p* < 0.001.

Figure S7 Percentage of CDR-H3s containing Y101 among sequences using RF1. The percentage of CDR-H3 sequences using RF1 that contained Y101 among selected B cell subsets in λ5KO versus wild-type (WT) mice. The *p*-value for the difference between follicular (FO) cells that achieved statistical significance (*p* = 0.01) is shown.

Table S1 List of unique non-repeated amino acid CDR-H3 sequences obtained from RT-PCR of V_H_7183-D-J-Cμ transcripts from sorted B cell subsets of λ5KO and wild-type (WT) BALB/c mice. A total of 563 unique λ5KO transcripts included 81 transcripts from fraction E, 90 from T1, 96 from T2, 77 from fraction F, 124 from follicular (FO), and 95 from MZ B cells. A total of 928 WT transcripts included 255 transcripts from fraction E, 102 from T1, 96 from T2, 254 from fraction F, 111 from FO, and 110 transcripts from MZ B cells.
