# Supplementary figures and images for: In the Absence of Central pre-B Cell Receptor Selection, Peripheral Selection Attempts to Optimize the Antibody Repertoire by Enriching for CDR-H3 Y101
Source: Front Immunol. 2018 Feb 7;9:120. doi: 10.3389/fimmu.2018.00120 (PMC5810287; doi:10.3389/fimmu.2018.00120)

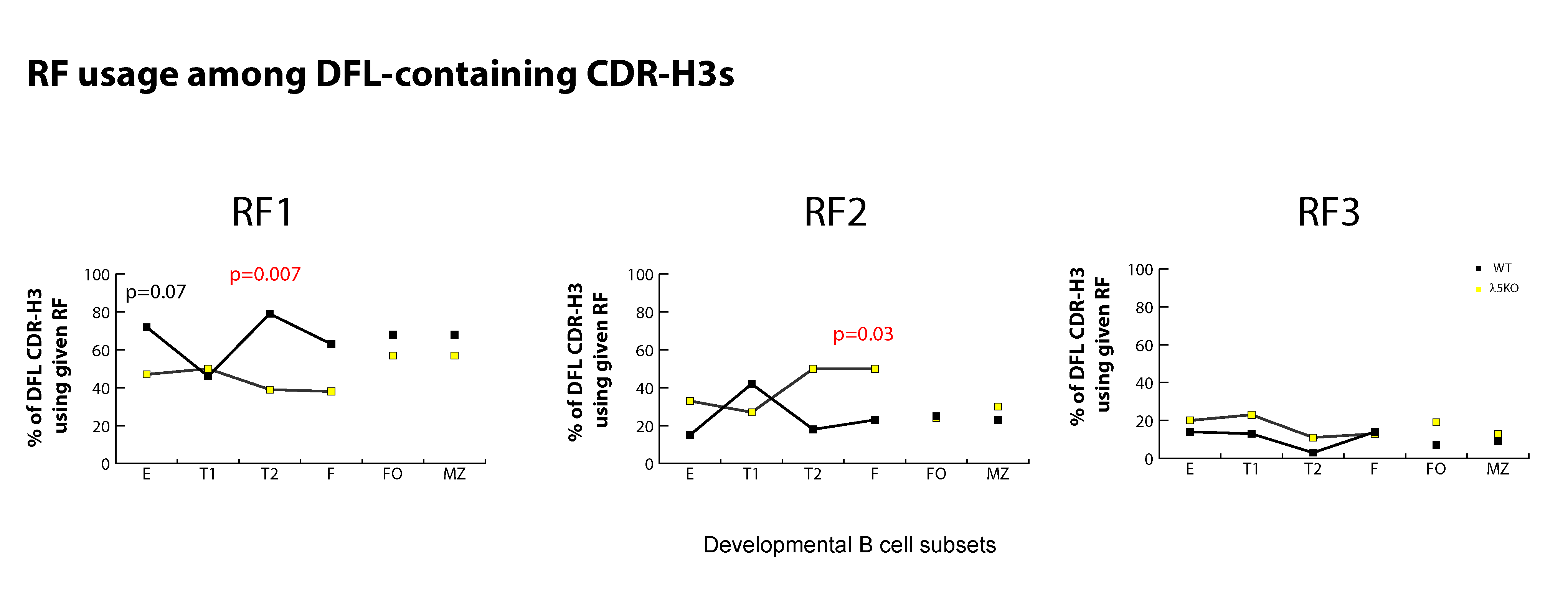

Supplement: Figure S2 — Differences in the use of DH DFL family reading frames (RFs) in CDR-H3s from λ5KO versus wild-type (WT). The percentage of RF usage in DFL containing CDR-H3 sequences obtained from λ5KO versus WT in the studied B cell subsets. The p-values for differences that achieved statistical significance (p ≤ 0.05) are shown. [file image_2.tiff]

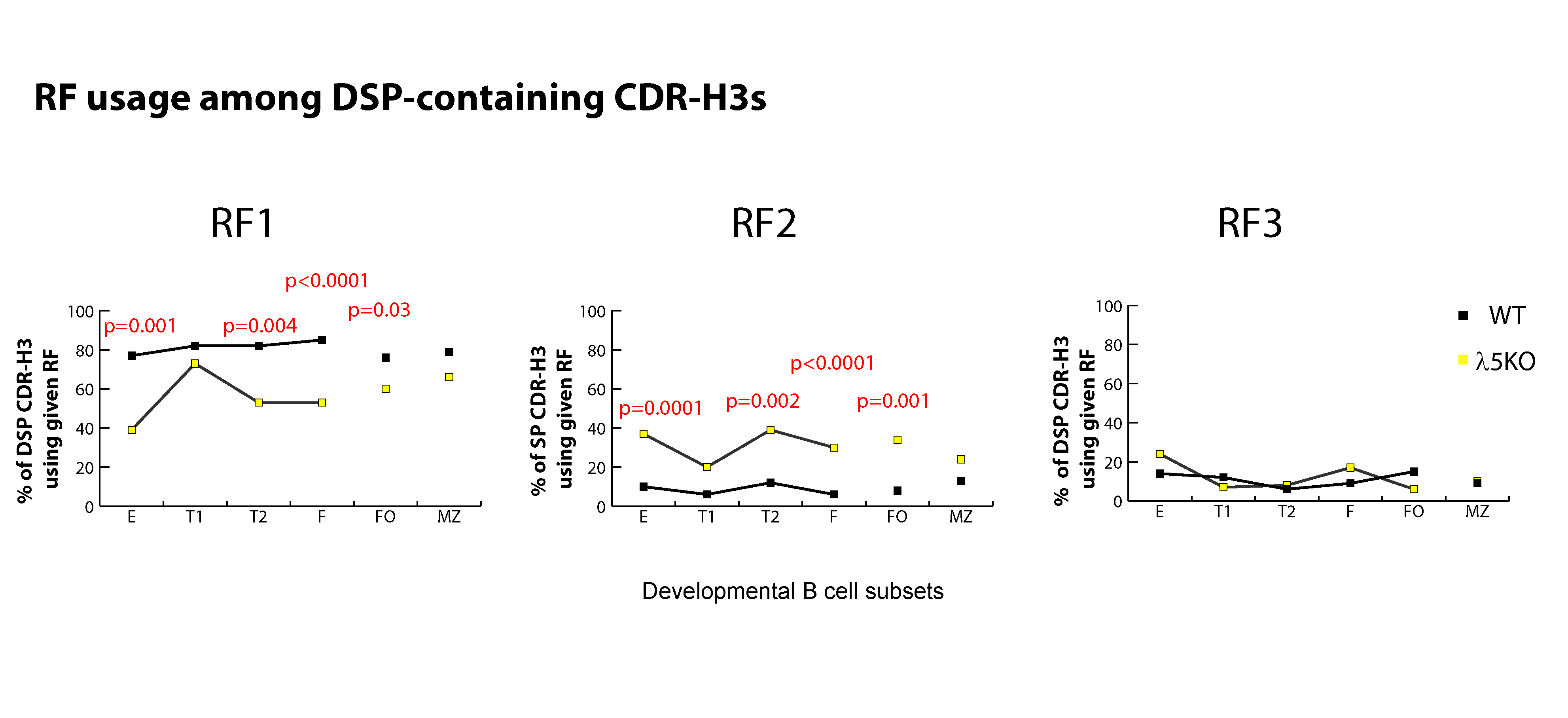

Supplement: Figure S3 — Differences in the use of DH DSP family reading frames (RFs) in CDR-H3s from λ5KO versus wild-type (WT). The percentage of RF usage in DSP-containing CDR-H3 sequences obtained from λ5KO versus WT in the studied B cell subset. The p-values for differences that achieved statistical significance (p ≤ 0.05) are shown. [file image_3.tiff]

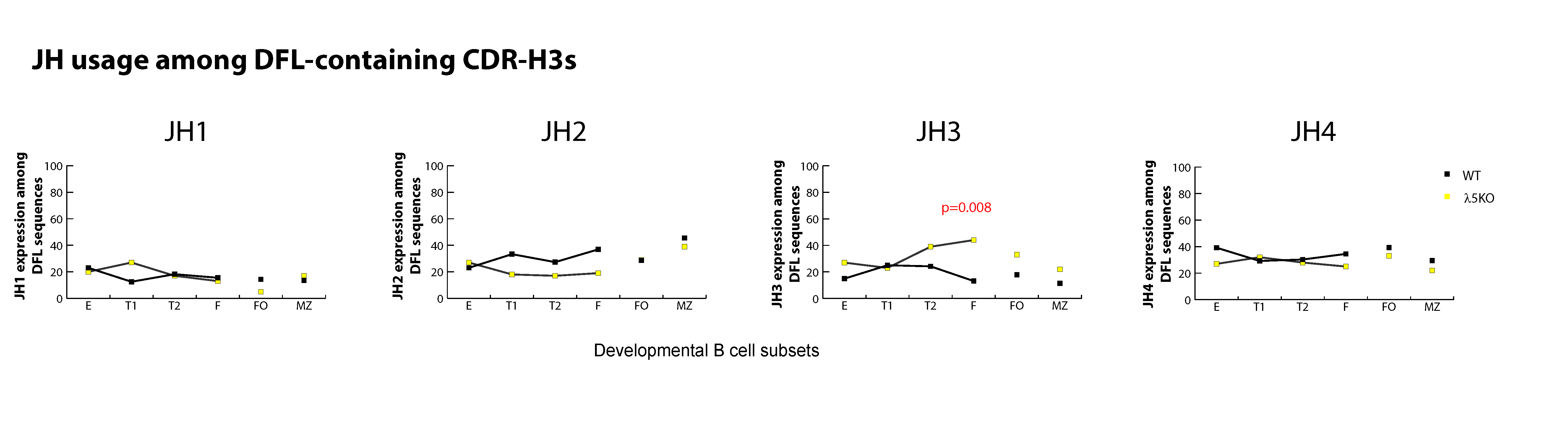

Supplement: Figure S4 — Differences in the use of JH in DFL family containing CDR-H3s from λ5KO versus wild-type (WT). The percentage of sequences from λ5KO versus WT using one of the four JH gene segments among CDR-H3s containing DH DFL gene segments among selected B cell subsets. The p-values for differences that achieved statistical significance (p ≤ 0.05) are shown. [file image_4.tiff]

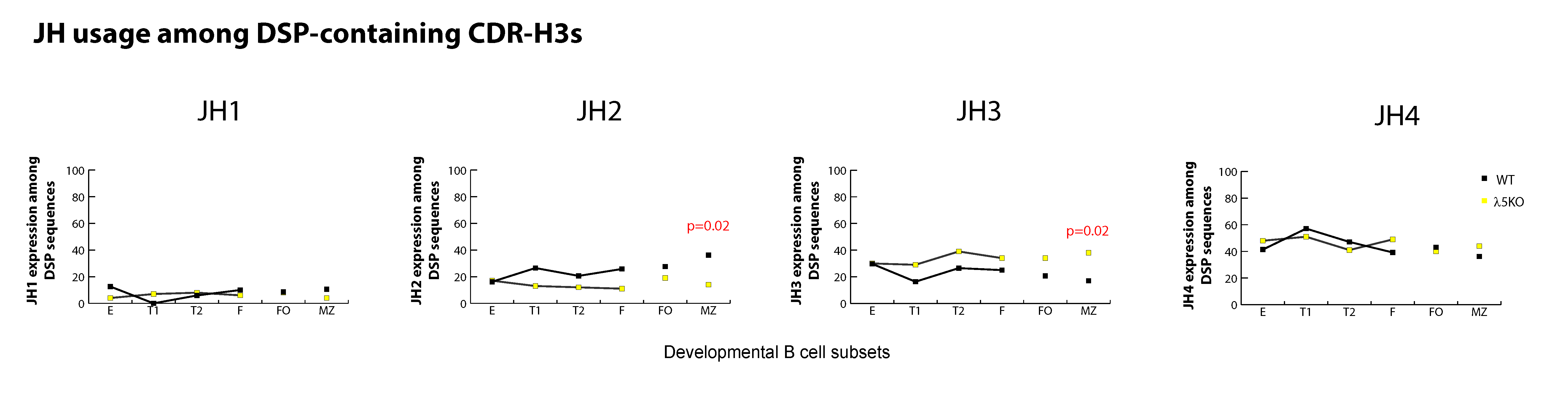

Supplement: Figure S5 — Differences in the use of JH in DSP family containing CDR-H3s from λ5KO versus wild-type (WT). The percentage of sequences from λ5KO versus WT using one of the four JH gene segments among CDR-H3s containing DH DSP gene segments from selected B cell subsets. The p-values for differences that achieved statistical significance (p ≤ 0.05) are shown. [file image_5.tiff]

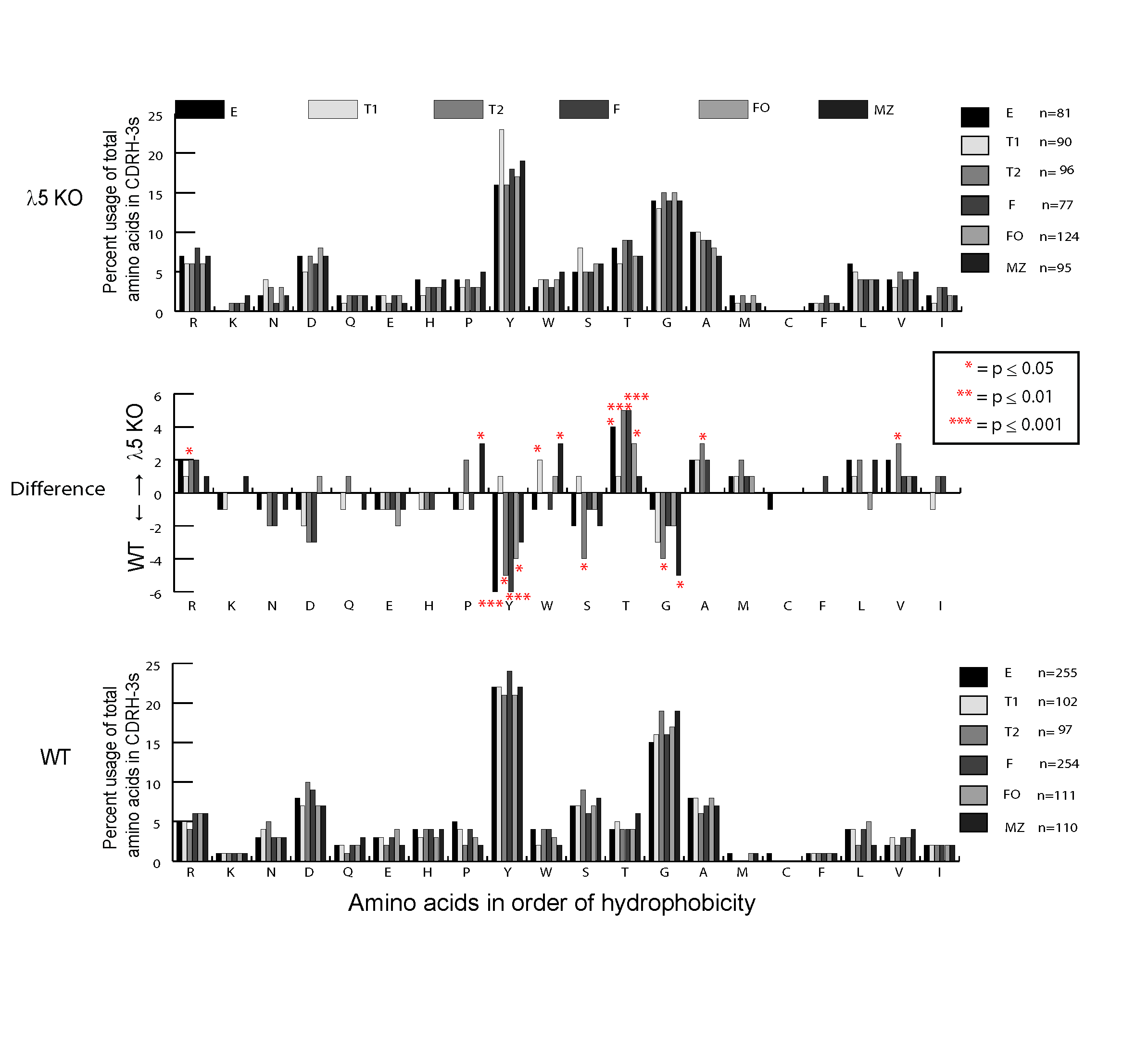

Supplement: Figure S6 — Distribution of individual amino acid usage in the CDR-H3s cloned from selected B cell subsets from λ5KO versus wild-type (WT). Upper panel, the percentage use of each individual amino acid, arranged in order of hydrophobicity, in the CDR-H3 loops of sequences cloned from selected λ5KO B cell subsets. Middle panel, the difference in percentage use of each amino acid in the CDR-H3 loops of λ5KO minus WT. Bottom panel, the percentage use of each individual amino acid, arranged in order of hydrophobicity, in the CDR-H3 loops of sequences cloned from selected WT B cell subsets. Significance values are listed as “*”, p ≤ 0.05; “**”, p ≤ 0.01; “***” and p < 0.001. [file image_6.tiff]

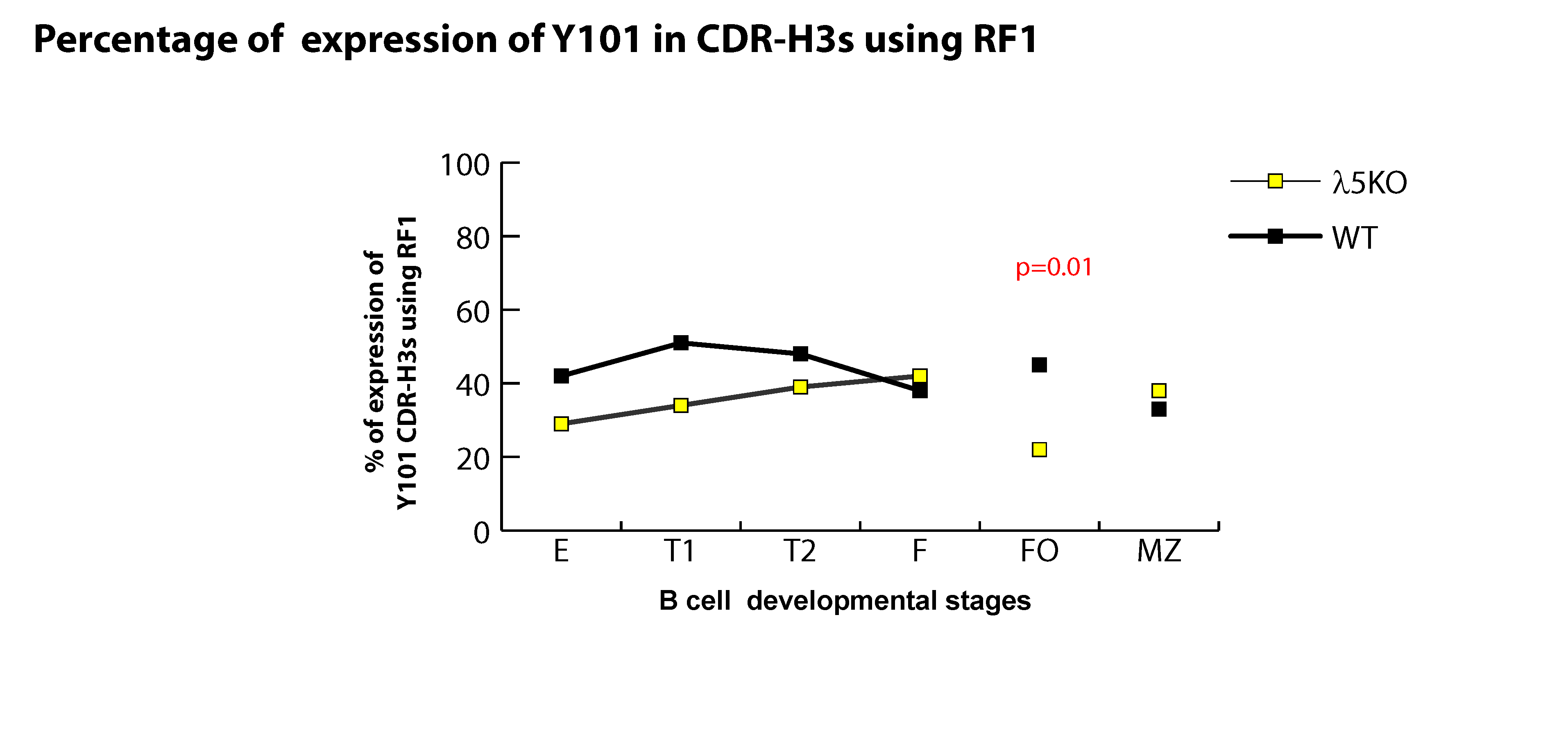

Supplement: Figure S7 — Percentage of CDR-H3s containing Y101 among sequences using RF1. The percentage of CDR-H3 sequences using RF1 that contained Y101 among selected B cell subsets in λ5KO versus wild-type (WT) mice. The p-value for the difference between follicular (FO) cells that achieved statistical significance (p = 0.01) is shown. [file image_7.tiff]
