# Supplementary material for: In the Absence of Central pre-B Cell Receptor Selection, Peripheral Selection Attempts to Optimize the Antibody Repertoire by Enriching for CDR-H3 Y101
Source: Front Immunol. 2018 Feb 7;9:120. doi: 10.3389/fimmu.2018.00120 (PMC5810287; doi:10.3389/fimmu.2018.00120)
Supplement: Table S1 — List of unique non-repeated amino acid CDR-H3 sequences obtained from RT-PCR of VH7183-D-J-Cμ transcripts from sorted B cell subsets of λ5KO and wild-type (WT) BALB/c mice. A total of 563 unique λ5KO transcripts included 81 transcripts from fraction E, 90 from T1, 96 from T2, 77 from fraction F, 124 from follicular (FO), and 95 from MZ B cells. A total of 928 WT transcripts included 255 transcripts from fraction E, 102 from T1, 96 from T2, 254 from fraction F, 111 from FO, and 110 transcripts from MZ B cells. [file table_1.PDF]

Genotype	B cell subset	CDR-H3
λ5KO	E	ARDGNY
λ5KO	E	ARHGGRDFAY
λ5KO	E	ARQDWYFDV
λ5KO	E	AREEENSFHFDY
λ5KO	E	ARDGDYGNYGAY
λ5KO	E	ASHDGGFAY
λ5KO	E	ARHPTRDY
λ5KO	E	ARGRGLRRDWFAY
λ5KO	E	ARDGRDYDRAAWFAY
λ5KO	E	ARDPGGNYPSY
λ5KO	E	ARQGPYYGNLDY
λ5KO	E	ARHKAYFDY
λ5KO	E	ARDNYAMDY
λ5KO	E	ARHRGLRRPPAMDY
λ5KO	E	ARHDYYGSSYFAY
λ5KO	E	ARQPNYYAMDY
λ5KO	E	ARHNRGSSSYYAMDY
λ5KO	E	ARDGGLRRWYFDV
λ5KO	E	ARHGGGNWYFDV
λ5KO	E	ARHGVRDYLDY
λ5KO	E	ARHLRDGY
λ5KO	E	ARQGYDYAWFAY
λ5KO	E	AREDYGSSYVNFGV
λ5KO	E	ARHGDYGAMDY
λ5KO	E	ARHPARARAMDY
λ5KO	E	ARLDDYAYY
λ5KO	E	ARGSRYYYAMDY
λ5KO	E	RGSSPSYWYFDV
λ5KO	E	ARGAYYRYDGAMDY
λ5KO	E	SRRLTGTHYNAMDY
λ5KO	E	ARGVRDSMDY
λ5KO	E	ARRGLRYYYAMDY
λ5KO	E	ARTPYGNYGAMDY
λ5KO	E	ARHEVADY
λ5KO	E	ARVPDYYDYAMDY
λ5KO	E	ARQRHSLLRLYWYFDV
λ5KO	E	ARRLTGTHYYAMDY
λ5KO	E	SRRLTGTHYYAMDY
λ5KO	E	ARGGTPYYYAMDY
λ5KO	E	ARRGLPYYYAMDY
λ5KO	E	ARQEVTTGYYAMDY
λ5KO	E	ARRLSTWGFAY
λ5KO	E	ARDPCYGSSGYAMDY
λ5KO	E	ARGGIYGKRAMDY
λ5KO	E	ARTADLGDYPYAMDY
λ5KO	E	SRRLTGAHYYAMDY

λ5KO	E	ARSFYYGSSWFAY
λ5KO	E	ARGWSGYAMDY
λ5KO	E	ARDGTTAVSYGYFDV
λ5KO	E	ARSPLYDAMDY
λ5KO	E	ARQGVTTGYYAMDY
λ5KO	E	ARDPTIGTAWFAY
λ5KO	E	ARHEILRAMDY
λ5KO	E	ARLPSSWFAY
λ5KO	E	AREGAMIGY
λ5KO	E	ARDALYGYDVIAY
λ5KO	E	ARPMMVTHWYFDV
λ5KO	E	ARIFYDGYCYY
λ5KO	E	ARQGVTTGCYAMDY
λ5KO	E	ARRDGGLLLWFAY
λ5KO	E	ARHEGVRVRLLAMDY
λ5KO	E	ARRFLYYAMDY
λ5KO	E	AFMVTEGFAY
λ5KO	E	ARQGMVTTDY
λ5KO	E	ARSMVTGGYYAMDY
λ5KO	E	ARGGPTMITFAY
λ5KO	E	ARAVRAYYAMDY
λ5KO	E	ARQVITT
λ5KO	E	ARHGGLLYAMDY
λ5KO	E	AHITTATWFAY
λ5KO	E	ARHLLYFDY
λ5KO	E	ARSMVTTFDY
λ5KO	E	ARGAMITTAWFAY
λ5KO	E	ARAITTVVDAWFAY
λ5KO	E	ARLGITSAMDY
λ5KO	E	ARPIPPG
λ5KO	E	ARLTTVVDY
λ5KO	E	AGVMITTAMDY
λ5KO	E	ARVFIGFDY
λ5KO	E	ATLTPY
λ5KO	E	ASMDY
λ5KO	F	ARHDLDY
λ5KO	F	ARHDNRDYFDY
λ5KO	F	ARRPFDY
λ5KO	F	ARREGHDEDYTMDY
λ5KO	F	ARHRYGDYFDY
λ5KO	F	ARQDRSHAMDY
λ5KO	F	AGHRENRNHSRSLYWYFDV
λ5KO	F	ARHRYDAMDY
λ5KO	F	ARRDGYWFAY
λ5KO	F	ARERDYGDYAMDY
λ5KO	F	ARYGYDEYFDY
λ5KO	F	ARENYRYDDGGYYYAMDY

λ5KO	F	ARRYPYWYFDV
λ5KO	F	ARGGDHSRSYYFDY
λ5KO	F	ARGYRYYFDY
λ5KO	F	ARHGDDGYYPGFAY
λ5KO	F	ARHGPPYYFDY
λ5KO	F	ARQYDYYYAMDY
λ5KO	F	ARAYYRYDGFAY
λ5KO	F	ARDRGLYYDYDGGFAY
λ5KO	F	ARQEYDYDAGAYFDY
λ5KO	F	ARGRYRYGYAMDY
λ5KO	F	ARQGDYYGSSSWFAY
λ5KO	F	ARHGSSFDY
λ5KO	F	ARHYGSSYGPFAY
λ5KO	F	ARQSSGYGYFDY
λ5KO	F	ARGNYDYGAWFAY
λ5KO	F	ARQGRYGGTTYYYAMDY
λ5KO	F	ARDYDGTGSYAMDY
λ5KO	F	ARQGLRHYYAMDY
λ5KO	F	ARDRRMVTTFAY
λ5KO	F	ARSRPSMGMDY
λ5KO	F	ARHETARATYYAMDY
λ5KO	F	ARGQENGLMVTKRDYAMDY
λ5KO	F	ARHRTTIGRAWFAY
λ5KO	F	ARDAGLRRYYAMDY
λ5KO	F	ARHELLRDYAMDY
λ5KO	F	ARVWGIRRQRAMDY
λ5KO	F	ARERASLRYLYWYFDV
λ5KO	F	ARDGYYFFDY
λ5KO	F	ARTMDY
λ5KO	F	ARGAYDTYYYAMDY
λ5KO	F	ARVEDGYYRTFYAMDY
λ5KO	F	ARQRLAWFAY
λ5KO	F	ARSGIAY
λ5KO	F	ARRMVRGDAY
λ5KO	F	ARHRSTMITTWFAY
λ5KO	F	ARGGIYGKRAMDY
λ5KO	F	ARGGIYGKRAMDY
λ5KO	F	ARQTTATGYAMDY
λ5KO	F	AREGSIGTTDAWFAY
λ5KO	F	ARGQGLLRYYYAMDY
λ5KO	F	ARSMVTTRWYFDV
λ5KO	F	ARSTMITTRGYYYAMDY
λ5KO	F	ARQKGFITTASY
λ5KO	F	ARDGVTSFAY
λ5KO	F	AREGYIGNYVGFAY
λ5KO	F	ARLPYGNYVGFAY
λ5KO	F	ARHGGIPAWFAY

λ5KO	F	ARIPYSGSYALDV
λ5KO	F	ARGPGYVAWFAY
λ5KO	F	ARPGITTSAFAY
λ5KO	F	ARRIFTTVPYYAMDY
λ5KO	F	ARVTTCWYFDV
λ5KO	F	ARSVTTATPDY
λ5KO	F	ARQGITTVVATTWFAY
λ5KO	F	AKFTTATFYAMDY
λ5KO	F	ARGGITTVVARGAWFAY
λ5KO	F	ARAPLWFYAMDY
λ5KO	F	ATTGTFLMDY
λ5KO	F	ARHLLLGPMDY
λ5KO	F	ARAGVTTLYAMDY
λ5KO	F	ARAGAVWFPFAY
λ5KO	F	ARGGMISAMDY
λ5KO	F	ARLVREAY
λ5KO	F	ARKALLLSFAY
λ5KO	F	ARVSALYAMDY
λ5KO	T1	ATRFAY
λ5KO	T1	ARDGNYDY
λ5KO	T1	ATKNYRYSYFDY
λ5KO	T1	ARGHYRWDY
λ5KO	T1	ASDSFDY
λ5KO	T1	ARQEDYDYDYYAMDY
λ5KO	T1	AVPKSFAY
λ5KO	T1	ARGRRDYAMDY
λ5KO	T1	ARSDRYDVRFAY
λ5KO	T1	ARQTQDAMDY
λ5KO	T1	ARRGNYDYDTDPSYAMGY
λ5KO	T1	ARDGNYWYFDV
λ5KO	T1	ARSERYDVNFDY
λ5KO	T1	ARDGYFAY
λ5KO	T1	ARHYDYDYYAMDY
λ5KO	T1	ARPRYEYYAMDY
λ5KO	T1	VYGNSFAY
λ5KO	T1	ARSRGWYFDV
λ5KO	T1	ARRYGYEAMDY
λ5KO	T1	ARKNPYDSSLFAY
λ5KO	T1	ATTTGRRYYAMDY
λ5KO	T1	ARSRYGNYAMDY
λ5KO	T1	ARGHYTWDY
λ5KO	T1	ARSHYGYVDY
λ5KO	T1	ARDYGNYAMDY
λ5KO	T1	ARDYGNYYAMDY
λ5KO	T1	ARHGSDGYYEAWFAY
λ5KO	T1	ARNYGSSYYFDY
λ5KO	T1	ARHEGPAWFAY

λ5KO	T1	ARPSMDY
λ5KO	T1	ARGDYYGSTSYWYFDV
λ5KO	T1	ARGNYPYYAMDY
λ5KO	T1	AREGVRRAWFAY
λ5KO	T1	ASQGEDGNYVGAMDY
λ5KO	T1	ARQGELGRAYYFDY
λ5KO	T1	ARYYGSSYWYFDV
λ5KO	T1	ARYYGSSYWYFDV
λ5KO	T1	ARETYYGNYLSWFAY
λ5KO	T1	ARAYGNSPFAY
λ5KO	T1	ARQYGNYVRGAMDY
λ5KO	T1	ARDRAATTGDYAMDY
λ5KO	T1	ARYGNFYWYFDV
λ5KO	T1	ARSGYGYADFDY
λ5KO	T1	ARDGGIYDGPWFAY
λ5KO	T1	ARRGNYPIYAMDY
λ5KO	T1	ARRGNYPIYAMDH
λ5KO	T1	TRRGGGAMDY
λ5KO	T1	ARYYYGSSYAMDY
λ5KO	T1	ARATNGYYYAMDY
λ5KO	T1	ARSGFDY
λ5KO	T1	ARSSWAYYFDY
λ5KO	T1	ARSYYGSSYPLFAY
λ5KO	T1	ARRGITSWFAY
λ5KO	T1	ARRGRPMMVTTGYYAMDY
λ5KO	T1	AGGGKD
λ5KO	T1	ARDPLYYYGSSYVFDY
λ5KO	T1	ARREGGLLSWFAY
λ5KO	T1	ARAPYFDY
λ5KO	T1	ASLRFAY
λ5KO	T1	ARANWGFWYFDV
λ5KO	T1	ASDFFDY
λ5KO	T1	ARFYYGSSYYAMDY
λ5KO	T1	ARGIWRYYAMDY
λ5KO	T1	ARGGGNYIFAY
λ5KO	T1	ARGLLRSYWYFHV
λ5KO	T1	ARHGTTVVDY
λ5KO	T1	ARFYEGAFDY
λ5KO	T1	ARGSLLRPYYAMDY
λ5KO	T1	ARTSTATFWYFDV
λ5KO	T1	ARGTTATLYWYLDV
λ5KO	T1	ARRWLLWYFDV
λ5KO	T1	ARDVDYAFYYAMDY
λ5KO	T1	ARGCLEHAWFAY
λ5KO	T1	ARQTLYAMDY
λ5KO	T1	ARAGNYVYYAMDY
λ5KO	T1	ARSRGTTVVYFDY

λ5KO	T1	ARAPPSMVTTWGAMDY
λ5KO	T1	ARAGGGSY
λ5KO	T1	ARHGPLLLHMDY
λ5KO	T1	ARGEMITAWFAY
λ5KO	T1	ARGLLLRPYYAMDY
λ5KO	T1	AKSSGYVLYYAMDY
λ5KO	T1	ARVGVNLYYYAMDY
λ5KO	T1	ARLLRYAMDY
λ5KO	T1	ASLTTFAY
λ5KO	T1	ASMVTTWFAY
λ5KO	T1	ARAMVNYAMDY
λ5KO	T1	ARAMVNYAMDY
λ5KO	T1	ARLAYGYVWFAY
λ5KO	T1	ARLTTATAMDY
λ5KO	T2	ARQRDWDFAY
λ5KO	T2	ARDGNMDY
λ5KO	T2	ARDRDAMDY
λ5KO	T2	ARGRNGNYDYFDY
λ5KO	T2	ARQGNYFDV
λ5KO	T2	VRQGNYFDV
λ5KO	T2	ARDWDYDYWFAY
λ5KO	T2	ARHGNYFDY
λ5KO	T2	ARHTHRYDAMDY
λ5KO	T2	ARRWWGGY
λ5KO	T2	ARRDYDYAMDY
λ5KO	T2	AREGFAY
λ5KO	T2	ARDKYGNYDAMDY
λ5KO	T2	ARYYYGSRKFAY
λ5KO	T2	ARDGNYWYFDV
λ5KO	T2	ARHQYGTPFAY
λ5KO	T2	ASYDYDQAWFAY
λ5KO	T2	ARDSYRYDAWFAY
λ5KO	T2	ARDSYRYDAWFAY
λ5KO	T2	TRGRGYDGFAY
λ5KO	T2	ASRYGPWFAY
λ5KO	T2	ARGRGGNYYFDY
λ5KO	T2	ARHDYYGSSWDWFAY
λ5KO	T2	ARRGPDSSGYPFAY
λ5KO	T2	ARHGYDYDDGAWFAY
λ5KO	T2	ASRPTGTLAY
λ5KO	T2	ARFYDYDEGFAY
λ5KO	T2	ARQTTARYFDV
λ5KO	T2	ARSRGNYPYAMDY
λ5KO	T2	ARDTGRYYYAMDY
λ5KO	T2	ARKSGGFAY
λ5KO	T2	AREMENYYAMDY
λ5KO	T2	ARGDTTFAY

λ5KO	T2	ARSRPYAMDY
λ5KO	T2	ARHPYYYGSSYGYFDV
λ5KO	T2	ARGGYEHYYAMDY
λ5KO	T2	ARDSHYYGSSYAFDY
λ5KO	T2	ARNYAMDY
λ5KO	T2	ARGYYRCYDY
λ5KO	T2	ARNYGSYYAMDY
λ5KO	T2	TRDLGGNYDAMDY
λ5KO	T2	ARLGRRYAMDY
λ5KO	T2	ARHEGLLRSLDV
λ5KO	T2	ARHPLGLRDYYAMDY
λ5KO	T2	ARGGKGAMDY
λ5KO	T2	ARSGDGYFSFDY
λ5KO	T2	ARRATAPYDY
λ5KO	T2	ARDEGSTMITRAWFAY
λ5KO	T2	ARGRVKGYAMDY
λ5KO	T2	ARSLKGWYFDY
λ5KO	T2	ARHGVTTDYYAMDY
λ5KO	T2	ARVYYGNPPYAMDY
λ5KO	T2	ARSCDGYYYAMDY
λ5KO	T2	ARTRAGYAMDY
λ5KO	T2	ARLGYGYDGFAY
λ5KO	T2	ARQKFITTATRFAY
λ5KO	T2	ARHGGIGTGRYAMDY
λ5KO	T2	ARHGGIGTGRYAMDY
λ5KO	T2	ARERGMITFAY
λ5KO	T2	ARGRTTVGFAY
λ5KO	T2	ARQAGYDGYYIGAMDY
λ5KO	T2	ARPPTIGTTWGYYYAMVY
λ5KO	T2	ARQPSMIPNAMDY
λ5KO	T2	ARGITNYFDY
λ5KO	T2	ARDRVVTYFDY
λ5KO	T2	ARGGGYLYYFDY
λ5KO	T2	ARHSMVTSWFAY
λ5KO	T2	ARQIGGYAWFAY
λ5KO	T2	ARLLWLRRGWFAY
λ5KO	T2	ARVPTIGTTWGYYYAMDY
λ5KO	T2	ARGIYYGNLGFAY
λ5KO	T2	ARQGIASWFAY
λ5KO	T2	ARGGVLRSWFAY
λ5KO	T2	ARPPTIGTT
λ5KO	T2	ARGPVPMDY
λ5KO	T2	ARMVTRGYAMDY
λ5KO	T2	ARRGVLPGFAY
λ5KO	T2	ARISKSAMDY
λ5KO	T2	ARGITTGWFAY
λ5KO	T2	ARTMVTKVNYAMDY

λ5KO	T2	ARHSLLYFDA
λ5KO	T2	ARHSLLYFDV
λ5KO	T2	ARHLITTSYAMDY
λ5KO	T2	AIITTGYAMDY
λ5KO	T2	ARHDLTTVVATAWFAY
λ5KO	T2	ARGSPTVVGYAMDY
λ5KO	T2	ARQLPVFAY
λ5KO	T2	ASMVTTWFAY
λ5KO	T2	ARRFLAMDY
λ5KO	T2	ARHVTTVVAAWFAY
λ5KO	T2	ARVMVTSYAMDY
λ5KO	T2	ASLLTAWFAY
λ5KO	T2	ARLVTTALDY
λ5KO	T2	ARLMVTGAMDY
λ5KO	T2	ATMVTTTY
λ5KO	T2	ARVEYY
λ5KO	FO	APRFAY
λ5KO	FO	AIRRFAY
λ5KO	FO	ARDEEY
λ5KO	FO	ATRSDFDY
λ5KO	FO	ARHDDYDYDGDY
λ5KO	FO	ARHNDYDETPFAY
λ5KO	FO	ARHEYDGNFDY
λ5KO	FO	ARHDGDYDYFDY
λ5KO	FO	ARQDKGYYDYDWFAY
λ5KO	FO	ARHYGDY
λ5KO	FO	ARQDRYGPWFAY
λ5KO	FO	ARRYDRYAMDY
λ5KO	FO	ARAHEEGREPWFAY
λ5KO	FO	ARRYGYDEGFAY
λ5KO	FO	ARHDGNDAMDY
λ5KO	FO	ARERGDYDVRYFDV
λ5KO	FO	ARDRMLTT
λ5KO	FO	ARRGYDYNDYAMDY
λ5KO	FO	ARDHYRYDGAWFAY
λ5KO	FO	ARDRYAMDY
λ5KO	FO	ARNGNYWYFDV
λ5KO	FO	ARERYRYAHAMDY
λ5KO	FO	ARGYGKDWFAY
λ5KO	FO	ARDYGNYDAMDY
λ5KO	FO	ARDYYGFAY
λ5KO	FO	ASYGNYKAY
λ5KO	FO	ARQYDYDGYAMDY
λ5KO	FO	ARQGWFAY
λ5KO	FO	ARPPAY
λ5KO	FO	ARSTGKEGYFDY
λ5KO	FO	ARDDYYAMDY

λ5KO	FO	ARQGTRGY
λ5KO	FO	ARRYGNYPYAMDY
λ5KO	FO	ARRGDYAWFAY
λ5KO	FO	ARRRVLRRGFAY
λ5KO	FO	ARDWGTEAY
λ5KO	FO	AIGTTDPYYFDY
λ5KO	FO	ARHLYYRYDDGAMGY
λ5KO	FO	ARQLESDGYPY
λ5KO	FO	ARPLYYRYPHWYFDV
λ5KO	FO	ARSWGNWFAY
λ5KO	FO	ARDYYGSDAMDY
λ5KO	FO	ARQGGTRYYYAMDY
λ5KO	FO	ARGDRYDAATTFH
λ5KO	FO	ARSPPYYDYDGGYAMDY
λ5KO	FO	ARHERGEGLYDGYLFAY
λ5KO	FO	ARQDYGSSWAMDY
λ5KO	FO	ARDWGGSSYFDY
λ5KO	FO	ARRSSYYAMDY
λ5KO	FO	AREYGNLYYFDY
λ5KO	FO	ARSISHYYGSSHKFAY
λ5KO	FO	ARDGNYLFAY
λ5KO	FO	ARHPRPTIGTTTYFDY
λ5KO	FO	ARHGGYDLYYFDY
λ5KO	FO	ARGRSAWFAY
λ5KO	FO	ARSGNSYYAMDY
λ5KO	FO	ARQGYGSSYVKFAY
λ5KO	FO	AREEDMVTTRGAMDY
λ5KO	FO	ARHGIYYGYDGGPFAY
λ5KO	FO	ARHESISYFDY
λ5KO	FO	AQSPAY
λ5KO	FO	AREFGGYYYFDY
λ5KO	FO	ARSWEVRRVMDY
λ5KO	FO	ARSTFDY
λ5KO	FO	ARSYRYDVEVGWFAY
λ5KO	FO	ARFGNPGYFDV
λ5KO	FO	ARRVDYYAMDY
λ5KO	FO	ARVPDYYGSSQAWFAY
λ5KO	FO	ARPAYYRYDVGYYAMDY
λ5KO	FO	ARDQGITTAY
λ5KO	FO	ARHFQTARAPAWFAY
λ5KO	FO	ARDGLLRYTRAMDY
λ5KO	FO	ARGGAYYKAWFAY
λ5KO	FO	ARQGGLRAFAY
λ5KO	FO	ARQGVTTRAVDY
λ5KO	FO	AREGGNYVAWFAY
λ5KO	FO	ARGDSSGYVWFAY
λ5KO	FO	ARSGYYAMDY

λ5KO	FO	ARAYGNYLKAMDY
λ5KO	FO	ARGHDLGGAMDY
λ5KO	FO	ARDPLYYYGMGYAMDY
λ5KO	FO	ARERGMIYYAMDY
λ5KO	FO	ARHGATWAMDY
λ5KO	FO	ARSTTAWFAY
λ5KO	FO	ARSYGGFGYFDY
λ5KO	FO	ARAVRYSWFAY
λ5KO	FO	ARGNTTVVDY
λ5KO	FO	ARHYGNYPLLCFDY
λ5KO	FO	ARGGRSAMVTTWYFDV
λ5KO	FO	TRDGYLAWFAY
λ5KO	FO	ARVGNSFAY
λ5KO	FO	ASLLRLRDYAMDY
λ5KO	FO	ARQGTMITPFAY
λ5KO	FO	ARGWAPMMVTTRGYFDY
λ5KO	FO	ARSGNFAMDY
λ5KO	FO	ARHPLIYWYFDV
λ5KO	FO	ARDGIGTYAMDY
λ5KO	FO	ARQTSMVMNYAMDY
λ5KO	FO	ARGGVTGYYYAMDY
λ5KO	FO	ARQAIGTTNFFDY
λ5KO	FO	ARHFMINWYFDV
λ5KO	FO	ARDSMITTGFAY
λ5KO	FO	ARSRFFRFAMDY
λ5KO	FO	ARGHMITTTMDY
λ5KO	FO	ARRGLLSWFAY
λ5KO	FO	ARSGSMVTTWFAY
λ5KO	FO	ARYAMDY
λ5KO	FO	ARSGSMMVTPYFDY
λ5KO	FO	ARQGFMVKGAIDY
λ5KO	FO	ARHLITWYFDV
λ5KO	FO	ARHAGGLWFYAMDY
λ5KO	FO	ARLATGTWFAY
λ5KO	FO	ARGMVNYAMDY
λ5KO	FO	ARSIGTTGAMDY
λ5KO	FO	ARGGCMDY
λ5KO	FO	ARGSLYAMDY
λ5KO	FO	ARGGTMITLYGFDY
λ5KO	FO	ARGGAITTVVATRTFAY
λ5KO	FO	ARALSLLRPAWFAY
λ5KO	FO	ARGSMVITLYYYAMDY
λ5KO	FO	ARPSLLLGYFDY
λ5KO	FO	ARVPLLYYAMDY
λ5KO	FO	ARGGAVVYFDY
λ5KO	FO	ARGRVITTVVAFFDY
λ5KO	MZ	ATRFAY

λ5KO	MZ	ARQRGNY
λ5KO	MZ	ARDKMDY
λ5KO	MZ	ARDEEY
λ5KO	MZ	ARSQDRYDGAY
λ5KO	MZ	ARHNGNYEDWYFDV
λ5KO	MZ	ARHDGNYDWFAY
λ5KO	MZ	AREGDGNYAAY
λ5KO	MZ	ARSQSRYYFDY
λ5KO	MZ	ARHDYDAMDY
λ5KO	MZ	ARGYYDYDFDY
λ5KO	MZ	ARRYGNSPWFAY
λ5KO	MZ	ARRDYGYKAMDY
λ5KO	MZ	ARDTGHYFDY
λ5KO	MZ	ARGNGNYLDY
λ5KO	MZ	ARSHGSRPYFDY
λ5KO	MZ	ARHSDGYYFDY
λ5KO	MZ	ARQGYYDWYFDV
λ5KO	MZ	ARRDYYAMDY
λ5KO	MZ	ANYYRSPWFAY
λ5KO	MZ	ARGKYGNYYFDY
λ5KO	MZ	ARSYYRYPWFAY
λ5KO	MZ	AGTKKAFAY
λ5KO	MZ	ARQGDGYYWFAY
λ5KO	MZ	ARQGDYGSSYDWYFDV
λ5KO	MZ	ARHAPYDYDDAMDY
λ5KO	MZ	ARAHYDDDYAMDY
λ5KO	MZ	ARRPDGYYVRFAY
λ5KO	MZ	ARHPPYDGYHYYAMDY
λ5KO	MZ	ARGWDYYFDY
λ5KO	MZ	ASYDYHYAMDC
λ5KO	MZ	ARHSPYDGYHYYAMDY
λ5KO	MZ	ARRDDGYYVLDY
λ5KO	MZ	ARHQWYAMDY
λ5KO	MZ	ARGEGGNYDYYAMDY
λ5KO	MZ	ASYGNTWFAY
λ5KO	MZ	ARHKAKAWFAY
λ5KO	MZ	ARHYGSSYYFDY
λ5KO	MZ	ARGRGGWFAY
λ5KO	MZ	ARHEYSLYRYDGYAMDY
λ5KO	MZ	ARDGYYRGSAMDY
λ5KO	MZ	ARHGYDYSYYAMDY
λ5KO	MZ	ARGPPYYFDY
λ5KO	MZ	ASPPVRRGTWFAY
λ5KO	MZ	ASPPVRRGTWFAY
λ5KO	MZ	ARHQKITTTTGY
λ5KO	MZ	ARHGNYFYWYFDV
λ5KO	MZ	ARRVPHYYGLDY

λ5KO	MZ	ARPYYYGSSPWFAY
λ5KO	MZ	ARWYFDV
λ5KO	MZ	ARSGYYVDY
λ5KO	MZ	ARSPTGHYAMDY
λ5KO	MZ	ARRELRLGFAY
λ5KO	MZ	ASNKLGGFAY
λ5KO	MZ	ARAYDGYDY
λ5KO	MZ	ARVGYYGSSSPRYFDV
λ5KO	MZ	ARHGSLGLRRFAY
λ5KO	MZ	ARDRTMIRGYYAMDY
λ5KO	MZ	ARRGNYPIYAMDY
λ5KO	MZ	ARGGGSSPFDY
λ5KO	MZ	ARSGLRSQAWFAY
λ5KO	MZ	ARRGLRLRAMDY
λ5KO	MZ	ARIYDGYYSWFAY
λ5KO	MZ	ASSGYDYAAMDY
λ5KO	MZ	ARGQGYYGYVYYAMDY
λ5KO	MZ	ARGWPAWFAY
λ5KO	MZ	ARGDYGLFAY
λ5KO	MZ	ARRVTGGYYAMDY
λ5KO	MZ	ARRGNCPIYAMDY
λ5KO	MZ	ARGTTAWFAY
λ5KO	MZ	ARELPYAMDY
λ5KO	MZ	ARHGTTVVDY
λ5KO	MZ	ARAWSRFAWFAY
λ5KO	MZ	ARDIGTFDY
λ5KO	MZ	ARQATIGTTDGVYYAMDY
λ5KO	MZ	ARGYYGLYAMDY
λ5KO	MZ	ARSTMIAY
λ5KO	MZ	ARRTTGICLEY
λ5KO	MZ	ARHEIFLDY
λ5KO	MZ	ARPPVRLLSWFAY
λ5KO	MZ	ARHAIGTTSAMDY
λ5KO	MZ	ARGALLGGRWFAY
λ5KO	MZ	ARQVGSTMITTGFAY
λ5KO	MZ	ARGKVLRLGFAY
λ5KO	MZ	ARHVTTVVTYWYFDV
λ5KO	MZ	TRGGVYGSISWFAY
λ5KO	MZ	ARFGNFAWFAY
λ5KO	MZ	ATLPTMDY
λ5KO	MZ	AKETTMITTVAMDY
λ5KO	MZ	ARGRTTVVLFDY
λ5KO	MZ	ARQAVVANFDY
λ5KO	MZ	ARSTVVATGTFDY
λ5KO	MZ	ARVAMVTRAMDY
λ5KO	MZ	ARPLLMVTPLYAMDY
λ5KO	MZ	ARGTTVVVLDY

WT	E	ARHDRAY
WT	E	ARHAAY
WT	E	ARHMDY
WT	E	ARQRDGLAY
WT	E	ARQRGYDERHYFDY
WT	E	ARQRGLDY
WT	E	ARHENWEPFAY
WT	E	ARQDGNRGFAY
WT	E	ARHDYDYDEWFAY
WT	E	ARKYFDV
WT	E	ARQNSRGFAY
WT	E	ARHEGNPFAY
WT	E	ARDKDYRYDDYAMDY
WT	E	AIHYDPFAY
WT	E	ARQNYYEDY
WT	E	ARHGRRDAMDY
WT	E	ARERDYYGNSFAY
WT	E	ARDPGKLYY
WT	E	ARHERWSYYFDY
WT	E	AGTDYRYDGFAY
WT	E	ARTDDWFAY
WT	E	ARHDYRSSWFAY
WT	E	ARDNYRYDYAMDY
WT	E	ARDRYGYDGFAY
WT	E	ARREDYYGSQRDYYAMDY
WT	E	ARHDYGNYNWYFDV
WT	E	ARHDGNYPWFAY
WT	E	ARHSNWDGFDY
WT	E	AARAYRYDYFDY
WT	E	ASYYDFDY
WT	E	ARHEGWFAY
WT	E	AREDYRYEGAMDY
WT	E	ARHEGGNWFAY
WT	E	ARYDYDWYFDV
WT	E	ARPESFDY
WT	E	ASHRYGGFAY
WT	E	ARRGNYPWYFDV
WT	E	ARQEGGKGYFDY
WT	E	ARRRGYGWYFDV
WT	E	ASDRYAMDY
WT	E	ARQENYPYAMDY
WT	E	AGHWYFDV
WT	E	AREKYGNFEYFDV
WT	E	ARPHDGYYYFDY
WT	E	ARRDGYSWYFDV
WT	E	ARSDGYYDFDV
WT	E	ARERAYYGNYEGVAY

WT	E	ARHGGYDGTY
WT	E	ARDHRYGYAMDY
WT	E	ARHEGWLRDFDY
WT	E	GNDYGFAY
WT	E	ARHGYGNYEAY
WT	E	ARYYDYDDYYAMDY
WT	E	ARWNRYDVKGSDY
WT	E	ARRNWAYFDV
WT	E	ARDRGYDEVWFAY
WT	E	ASREVYDYFAY
WT	E	ARDPNYYGSSYPPWFAY
WT	E	ARQGHYYGSSWDWYFDV
WT	E	ARHGGGEYYFDY
WT	E	ARQDWPYAMDY
WT	E	ARWQPRAGMDY
WT	E	ARHEGDYGNSFPWSAY
WT	E	ARHPPYGYDGAHFDY
WT	E	ASRTDYGNTGAMDY
WT	E	ASPHYYGRYYAMDY
WT	E	ASHYGYDEGYYATDY
WT	E	AREVRRDGAFDY
WT	E	ARGEGFAY
WT	E	TRDYYESAMDH
WT	E	ASYGNYSYYFDY
WT	E	ARPADEGYFDY
WT	E	AKPYYGSRDYYAMDY
WT	E	ARHGRYSYAMDN
WT	E	ARHRGYSYAMDY
WT	E	ARDGYYDPAWFAY
WT	E	ARHGNYPAWFAY
WT	E	ARYFDY
WT	E	ARHYYGSSFDY
WT	E	TRDFHYFAY
WT	E	TRDFHYFAY
WT	E	ARGYDYDAWFAY
WT	E	ARGQXYRYPYXAMDY
WT	E	ARDRITTARDWYFDV
WT	E	ARSRKIDYYGSRAPYWYFDV
WT	E	ARRAMDY
WT	E	ARDYYGSRGYYAMDY
WT	E	ARHYYGSSYWFDY
WT	E	ARHSYYDYDAAMDY
WT	E	ARLPRYDNAMDY
WT	E	ARGRAYGNYDYYAMDY
WT	E	ARHQIYYGNPWFAY
WT	E	ASQGGGYYFDY
WT	E	ARQGYDGAMDY

WT	E	ARANYYGSSYNWYFDV
WT	E	ARHYYGYGDAMDY
WT	E	ARHDYYGSSYAMDY
WT	E	ARDTWEGAMDY
WT	E	ARQRESGITWFAY
WT	E	ARQRLYYDYDVFDY
WT	E	ARHDGYGYAMDY
WT	E	TKQRTGTGAMDY
WT	E	ARPDYYAMDY
WT	E	ASGNYYGSSYDYYAMDY
WT	E	ARLHYGNPPYFDY
WT	E	ARSPGVRRGYYFDY
WT	E	ARHIGNHWYFDV
WT	E	ARHHYYGSINWYFDV
WT	E	ARDGDYDGGSLMDY
WT	E	ARQGDGNYVSWFAY
WT	E	ARHGTGTWAY
WT	E	ARWRYDVGYFDV
WT	E	ARHDYYGSFSMDY
WT	E	ARPDGYYVDWYFDV
WT	E	ARFGYPRYWYFDV
WT	E	TRSYYGSSPYWYFDV
WT	E	ARDRRYGSSYVGYAMDY
WT	E	ARDPYYGSSYVDWYFDV
WT	E	ARTPSLAY
WT	E	ARHPHYYGSSSLYWYFDV
WT	E	ARGPWDY
WT	E	ARHGRYYGNYVYAMDY
WT	E	ARSVYYGNYEWYFDV
WT	E	ARDYYGSPAWFAY
WT	E	ASLYYGNYEGVDY
WT	E	ARSPSSMDY
WT	E	ARGYYGYWYFDV
WT	E	ARHAYYYGSSPPDY
WT	E	ARQSYYYGSSPAWFAY
WT	E	ARHDWDLYAMDY
WT	E	ARHCPYYFDY
WT	E	ARGGSYYGNPYYYAMDY
WT	E	AGDYYGSSYAMDY
WT	E	AREGSYYGYVNWYFDV
WT	E	AREYGYAWFAY
WT	E	ARHGPIYYGSTPWNFDV
WT	E	ARDPYGSSYGAMDY
WT	E	ASGNPAWFAY
WT	E	ARDCSYYGPFAY
WT	E	ARHGVGRYFDY
WT	E	ARHGSSPYAMDY

WT	E	ARGPDSAFAY
WT	E	ASPAYYGNYTSPYYAMDY
WT	E	ARSPYGSSPELYYLDY
WT	E	ANYYGSSPGGFAY
WT	E	ARYHGSSYAMDY
WT	E	ARAYDGYGGWYFDV
WT	E	ARHSPGTAHYAMDY
WT	E	ARHSSGPAWFAY
WT	E	ARHEGLLRPFAY
WT	E	ARRVYYYGSSYFDY
WT	E	ARHHYSTATAMDY
WT	E	AREALRSSDYAMDY
WT	E	ARERGPTMINYYAMDY
WT	E	ARCGYDGWYFDV
WT	E	ARQGGYAMDY
WT	E	AKGGYWFAY
WT	E	ARFYYYGPYWYFDV
WT	E	ARQRQLGLFDY
WT	E	ARDYDGYLYAMDY
WT	E	ARDCSSYGFAY
WT	E	ARTEDY
WT	E	ARGGDYGNFYAMDY
WT	E	ARVYDYADYFDY
WT	E	AREKGNYGFFYAMDY
WT	E	ARETSYYYGSSYSLYYAMDY
WT	E	ASWGRAY
WT	E	ARGGSDATDY
WT	E	ARPLDGYYAY
WT	E	ARHAGRDY
WT	E	ARLDYYLDY
WT	E	VRGWGFAY
WT	E	ARRGITKGYAMDY
WT	E	ARLDGYYGFY
WT	E	ARHEGVPYAMDY
WT	E	ARDIYYYGSSSSYYAMDY
WT	E	ARRGLIYDGYPWFAY
WT	E	AREGVNPAWFAY
WT	E	ARGGYPYAMDY
WT	E	TRVGDGYEGYAMDY
WT	E	ARKRVALDY
WT	E	ARVLRPSDYFDY
WT	E	ARGGVRPFDY
WT	E	ARGTTATYYFDY
WT	E	ARLGWLRRRNY
WT	E	ARSHYYGSSLYAMDY
WT	E	ARHAYYAMDY
WT	E	ARTDGFGFAY

WT	E	ARIWYPGDYYAMDY
WT	E	ARRGITGYFDY
WT	E	APPTIGTTQFAY
WT	E	AREGWLHAMDY
WT	E	ARGKGYYCAMDY
WT	E	ARQVGIYYGYDNAMDY
WT	E	ARGIYYGNYPN
WT	E	ARHLPPDY
WT	E	ARGRVHYYGCYAMDY
WT	E	ARGNYGYLYAMDY
WT	E	ARAIDGYYEAWFAY
WT	E	ASIYYGNYAVDY
WT	E	ARDRRLLLFAY
WT	E	ARSLRYAMDY
WT	E	ARCTRAMDY
WT	E	ARQGLRLFDY
WT	E	ARKTIPYAMDY
WT	E	ARLGDYYAMDY
WT	E	ARGSGTGAMDY
WT	E	TRSVWSYHAMDY
WT	E	ARLYYGYVDYYAMDY
WT	E	ARLPICGRDYFDY
WT	E	ATLLRPPFAY
WT	E	ASLYGSHAMDY
WT	E	ARHALYYGYAMDY
WT	E	ARGAGTGFAY
WT	E	ARQGIYYAMDY
WT	E	ARGEVGFAY
WT	E	ARQGWLLRAMDY
WT	E	ARNDATMITGYAMDY
WT	E	ARHSLLREGLAMDY
WT	E	ARTGSSFYAMDY
WT	E	AREATATAMDY
WT	E	ARTPLITKTPPLFDY
WT	E	TLHYYGYVCAMDY
WT	E	AREANLLPYAMDY
WT	E	ARGLYYGSSYLYAMDY
WT	E	ARGGYGSSLLYFGV
WT	E	ARKVFDY
WT	E	ASLIYYDYDVAWFAY
WT	E	ARLLYGSSYGGDY
WT	E	AKWLLRGYAMDY
WT	E	ARPEISTMITTSDY
WT	E	ARSPLYAMDY
WT	E	ARPSKFITTVVARGFAY
WT	E	AREGFTTVVAKMDY
WT	E	ARPSITGAMDY

WT	E	ARGLRGFAMDY
WT	E	ARGFTMDY
WT	E	ARDCFFAY
WT	E	ARDGVLFPWFAY
WT	E	ARALLHWYFDV
WT	E	ARSMITTGFAY
WT	E	AREFITTVVADWYFDV
WT	E	ARDLAPRY
WT	E	ASPSMITTAY
WT	E	ARGSTMITLYAMDY
WT	E	TSTTVVAP
WT	E	ASGLVSPSDY
WT	E	ARLGAWFAY
WT	E	ARSMMVTIDY
WT	E	AELGQDY
WT	E	ARILMITYAMDY
WT	E	TRLLFTADV
WT	E	ASLLLF
WT	E	QMIH
WT	E	TRGYH
WT	F	ARRDDDLDY
WT	F	ARHEQYYFDY
WT	F	ARHEWGDY
WT	F	AKHPFDY
WT	F	ASHYDYDDDDGGFAY
WT	F	ARDYGAY
WT	F	VTDYGNYRFAY
WT	F	ARDHGYDEAY
WT	F	ARKGYDGHSRRYFDV
WT	F	ARHEDYRAMDY
WT	F	ARQGYDYDGDY
WT	F	ARQGPYYRYDDDGLDY
WT	F	ARDRRYYGGFAY
WT	F	ARQGPRYFDV
WT	F	ARHHDSYYFDY
WT	F	ARHEGDGYSY
WT	F	ARRPYDSPMDY
WT	F	ARQNWDNAMDY
WT	F	AREDGYWAY
WT	F	ARQRGGFAY
WT	F	ARDSGDY
WT	F	ARHDYDRAWFAY
WT	F	ARHRYDAMDY
WT	F	ARHGDYFDY
WT	F	ARRRYDVKYFDV
WT	F	TRDNDDLFDY
WT	F	ARQGYRYDGDWYFDV

WT	F	AREGRYEDYAMDY
WT	F	ARRGEYGNGFDY
WT	F	TRDQNYGSSYGY
WT	F	ARRSYYGNFDY
WT	F	ARERGYYGSNLDY
WT	F	ARRDGYDDYYAMDY
WT	F	ARQREYYGNYRVFAY
WT	F	ARGHYRWYFDV
WT	F	AREYDGSFAY
WT	F	ARRGYGNYFDY
WT	F	ARQRGRGAMDY
WT	F	ARDRPYGNYEGRGYAMDY
WT	F	TRDEGMDGYYRFPY
WT	F	ARDRGGPWYFDA
WT	F	ARTGRYDGFDY
WT	F	ARHEDYYGYGTDWYFDV
WT	F	ARHGGGRFAY
WT	F	ARQNYGYWYFDV
WT	F	ARYRYDAMDY
WT	F	ARDQDYGAMDY
WT	F	ARHKSDYYAMDY
WT	F	ARYYDYDGSMDY
WT	F	ARQGYYGRTFAY
WT	F	ARSRGLRRYFDY
WT	F	AREGTTHFDY
WT	F	ARHEAYYRYGLDY
WT	F	ARHSGDDYDAWFAY
WT	F	ARQKYGNYGDYYAMDY
WT	F	ARSRGLYYDYDRYFDY
WT	F	ARRGGYGSRYFDY
WT	F	AGHNDGYYGDYAMDY
WT	F	AIRYDPYYAMDY
WT	F	ARRNYGSSYWYFDV
WT	F	ARHGNRYGAMDY
WT	F	ARERGYGNYAMDY
WT	F	TREELYEEAY
WT	F	ARHGGDGNYDAMDY
WT	F	ARHERGWLSHFDY
WT	F	ARDPYYGSRRGAWFAY
WT	F	ARHNYGSPDAMDY
WT	F	ARHGDGYPYWYFDV
WT	F	ARDGRDGYYAWFAY
WT	F	ARDYGSSHWYFDV
WT	F	ARDYYGRAMDY
WT	F	ARHDDYDGGLFAY
WT	F	ARDGRYYAMDY
WT	F	ARHTYYYGSSYHDSWFAY

WT	F	ARGYDYDGTWFAY
WT	F	ARRTTGYFDY
WT	F	ARADDYWFAY
WT	F	ARHYYGYYFDY
WT	F	ARYYYGHYFDY
WT	F	AREGDPYYYGSSRAMDY
WT	F	ASPYGGY
WT	F	ARPVHYYDRYWYFDV
WT	F	ARWGNSFDY
WT	F	ARDGNYPYAMDY
WT	F	ARNLYDYEGFAY
WT	F	AREGPYGMRFAY
WT	F	ARPPWFAY
WT	F	ARGDYYGYVDY
WT	F	ARGYDGYYFDY
WT	F	ARHPYDYDVSFDY
WT	F	ARQNRYDVSYAMDY
WT	F	ARDYYGNYYAMDY
WT	F	ARHPDGYYYYAMDY
WT	F	AREDRFYAMDY
WT	F	ARDYYGSSYGDY
WT	F	ARPYDYDGAWFAY
WT	F	ARRDSSGYYYAMDY
WT	F	ARSGYYGNYPLDY
WT	F	ARDYYYGSSPYWYFDV
WT	F	ARDDGYYVNYFDY
WT	F	ARWDYDGAMDY
WT	F	ARHYYYGSRTYYAMDY
WT	F	ARQVYYDYDGWYYFDY
WT	F	ARPHYDYDVGWFAY
WT	F	ARQLRPSYWYFDV
WT	F	ALYDGPSTGYFDY
WT	F	ARHGNSYAMDY
WT	F	ARDGSSYEYYAMDY
WT	F	ARQDYYGSSYAMDY
WT	F	ARDDGYYGAMDY
WT	F	ARGDYGNAMDY
WT	F	ARDRPYGSSFWFAY
WT	F	ARQDYYGNYVGFDY
WT	F	ARERGGYGYDVLDY
WT	F	ASSYRYAFAY
WT	F	ARHRGGYGAMDY
WT	F	ARHGEGYYAMDY
WT	F	ARCYDYDGSMDY
WT	F	ARQGKLGEGFAY
WT	F	AKEVRRSYAVDY
WT	F	ARQLYRGFAY

WT	F	ARHGNWDVWFAY
WT	F	ARHGDGYYSYAMDY
WT	F	ARDFIGRYRYDGDYYAMDY
WT	F	ARHDLYDGYYSSTGYFDV
WT	F	ARHGDYEGVWFAY
WT	F	ARETQTYDYDVGAMDY
WT	F	AKEGGYYYGSSGYYFDY
WT	F	AREKALWRHYAMDY
WT	F	ARGGDYGYGWFAY
WT	F	ARDYYGSRVWYFDV
WT	F	ARPSNVDYFDY
WT	F	ARPYGNYAMDY
WT	F	AGQGDGYFFDY
WT	F	ARHHYCGSSLDY
WT	F	ARDEVRGYYAMDY
WT	F	ARGDYRYGVDYYAMDY
WT	F	ARHPSYYGYAMDY
WT	F	AREGPSYYAMDY
WT	F	ARDRSYGSSSLSWYFDV
WT	F	ARHPAMDY
WT	F	ARRGGYYAMDY
WT	F	ARHTLYGNYWYFDV
WT	F	ARYYYGSSYGY
WT	F	ANYGYWFAY
WT	F	ASIYYDYDGFAY
WT	F	ARDPPIYPYYFDY
WT	F	ARSGYYGSSYDYYAMDY
WT	F	ARRGGSSYGYYAMDY
WT	F	ARDGNYVDYAMDY
WT	F	ARSYYGEAWFAY
WT	F	ARHSYDGYVWFAY
WT	F	ARGGGDGYPYYAMDY
WT	F	ARQTLYGKGGFDY
WT	F	ARGGTTVKKYFDV
WT	F	AKDYYGGAMDY
WT	F	ATYGSSLAY
WT	F	ARRLYYYGHAMDY
WT	F	ARGPGSSVDY
WT	F	ARSLGY
WT	F	ARCFYDYDGYFDY
WT	F	ARQGYGINFDY
WT	F	TRRYGYLPDY
WT	F	ARHATSFDY
WT	F	ARGAAYYRYLDY
WT	F	ARHGDGYYLYYAMDY
WT	F	ARPGIYYDYDGSAWFAY
WT	F	ASGADTFAY

WT	F	ARRAYSYAMDY
WT	F	ARGDAMDY
WT	F	ARSTQLAHYWYFDV
WT	F	ARKGSLYWYFDV
WT	F	AREGNFYAMDY
WT	F	ARGGDWGAWFAY
WT	F	AAYDYVWYFDV
WT	F	TRYPLYRSYAMDY
WT	F	AREGITRYYAMDY
WT	F	ARPGYGYYAMDY
WT	F	ARQPYYYGSSYLAMDY
WT	F	ARHGYYVNYAMDY
WT	F	AREVGYSYYFDY
WT	F	ARRGITRGAWFAY
WT	F	ARASPGYYFDY
WT	F	ARDPYYGNNFVGAMDY
WT	F	ARQRGLTTATDY
WT	F	ARRRLRGIYAMDY
WT	F	ARGLYGYDWFAY
WT	F	ARHIYDGYPFYFDY
WT	F	ARGGFKFSYGSSPYYFDY
WT	F	ARQGLPYNYGSSLYYYAMDY
WT	F	ARGGGLLWYQRSYFDY
WT	F	ARPQSTAGTLYWYFDV
WT	F	AREGYDVAWFAY
WT	F	ARRGMVTTWSGYYFDY
WT	F	AREGWLLPRYYAMDY
WT	F	ARRVWSPYAMDY
WT	F	AREGLYPPAMDY
WT	F	ARHSYLFDY
WT	F	ATLWDWFAY
WT	F	ARRGPNFPITTATYSYAMDY
WT	F	ARHNITTVVENWYFDV
WT	F	ARQPGYLYAMDY
WT	F	ARGLRLPDYYAMDY
WT	F	ARGYGYLYYFDY
WT	F	ARWGVRLRYYAMDY
WT	F	ARSGWYAMDY
WT	F	AREGLPSYAMDY
WT	F	ARGRDLLLRSFDY
WT	F	ARGGLNSFAY
WT	F	ARHGGWGLFDY
WT	F	ARGDYYGSILYYFDY
WT	F	ARQVLGHWYFDV
WT	F	ARHGPLYAMDY
WT	F	ARPAIYYGSPWFAY
WT	F	ARITLDYGDFDY

WT	F	ARGYGSYGNFGVGTY
WT	F	ARIDGYSYAMDY
WT	F	ARHLPYAMDY
WT	F	ARETPTMITTGYAMDY
WT	F	ARGALRYSYAMDY
WT	F	ARQGIHYYGYVGAMDY
WT	F	ARWSYRLLGGFAY
WT	F	ARGWLLKNYAMDY
WT	F	ARLNWDVGFAY
WT	F	ARLYYGYLYWYFDV
WT	F	ARHGATITGYFDV
WT	F	SLATATGGGYFDY
WT	F	ARHQLGFYAMDY
WT	F	ARGGFPITRYAMDY
WT	F	ARLFYYYGSSYGAMDY
WT	F	ARTGTGLYYAMDY
WT	F	NMITTRAFAY
WT	F	AVGNLAWFAY
WT	F	ARPITATWYFDV
WT	F	ARQVTTVVAKGSWYFDV
WT	F	ARPLREVFFAY
WT	F	ARQTLITTGFDY
WT	F	ARGGPGISAY
WT	F	AREGVGAMDY
WT	F	AREAINSLFAY
WT	F	ARVGYGNLFAY
WT	F	ARWGITTASFDY
WT	F	NRESVLPNVFDV
WT	F	ARLMYDYAAMDY
WT	F	ARVGPSVLRYFDV
WT	F	ARHETITTVVVYYFDY
WT	F	ARWLLPYYAMDY
WT	F	ARHPITTVVADAMDY
WT	F	ARVLLRLRSPFFDY
WT	F	AIITTVYYFDY
WT	F	ARHGITTVVATGFDY
WT	F	ARGITTVTAMDY
WT	F	ARITTVPFDY
WT	F	ASLITTVVDY
WT	T1	ARNWDY
WT	T1	ARNRSMDY
WT	T1	ARRGDYDD
WT	T1	ARRDYRSYFDY
WT	T1	ARHGNYDY
WT	T1	ARRGRQEGGGNYFDY
WT	T1	AREGYDGDY
WT	T1	ARESFAY

WT	T1	ARHQDYGSSYDFDY
WT	T1	ARHEWDVRNYFDY
WT	T1	ARWEKDAMDY
WT	T1	ASTYYRYEEGYFDY
WT	T1	ARNPYDYDDGDYAMDY
WT	T1	ARRKYGKEGYAMDY
WT	T1	AREGGYYRYDRGAMDY
WT	T1	ARRGPWYFDV
WT	T1	ARHEVRRDYYAMDY
WT	T1	ARYYRYDKGYYAMDY
WT	T1	ARPDGYYFDY
WT	T1	ARDNYAMDY
WT	T1	ASPVDY
WT	T1	ARHPYYDYDSYAMDY
WT	T1	ARQKAYDYDGGDAMDY
WT	T1	ARHDYDGYYYAMDY
WT	T1	ARQPYGNYGGFAY
WT	T1	AREGNYEGAMDY
WT	T1	ASSYDYDEGYAMDY
WT	T1	ARQEYYRYDVENFNYYAMDY
WT	T1	TREGGPVDY
WT	T1	ARDHYYGSSSYYFDY
WT	T1	ARSNYGYFDY
WT	T1	ARGDYYGPFAY
WT	T1	ARHRGYAWFAY
WT	T1	ARHDSYAMDY
WT	T1	ARTGDYGNPGGYFDY
WT	T1	ARPYDYDGAWFAY
WT	T1	ARRRLTSYFDY
WT	T1	ARLTYDYDEGMDY
WT	T1	ASGGYERWYYAMDY
WT	T1	ARDYYGSSYWYFDV
WT	T1	AMGDYGSSYPFAY
WT	T1	PRQGGNYPSYAMDY
WT	T1	ARSGEHAMDY
WT	T1	ARPNPNYYGSSGAMDY
WT	T1	ARGNYEGAMDY
WT	T1	AIPYYRYDVNYAMDY
WT	T1	ASSYYRYDVYFDY
WT	T1	ARQTGTGYFDV
WT	T1	ARQLRGMDY
WT	T1	ARRQGLGYYFDY
WT	T1	ARSYYYGYFDY
WT	T1	ARRRFAMDY
WT	T1	ARRTTAKAMDY
WT	T1	ARRGAMDY
WT	T1	ARHGGNSGAMDY

WT	T1	ARHDYGNLYAMDY
WT	T1	ARQGTGTPYYYAMDY
WT	T1	ASPSSDGAMDY
WT	T1	ARGPFYYYGSRGGFAY
WT	T1	AREGQYGNYVSAMDY
WT	T1	ARGGYYFDY
WT	T1	ARALYYDYDNYAMDY
WT	T1	ARHGPPIYRYDVGSSLDY
WT	T1	ARGGYDGYAMDY
WT	T1	ARQGLYYGNHPYVMDY
WT	T1	ARSDSSGSAWFAY
WT	T1	AKTGTGPMDY
WT	T1	ARRITTPHYYAMDY
WT	T1	ARDSSGAMDY
WT	T1	ARPDLHYAMDY
WT	T1	ARQIYYGSSYYFDY
WT	T1	ARGDGSSLTYFDV
WT	T1	ARDYGYYSLYAMDY
WT	T1	ASKLTGSYYYAMDY
WT	T1	ARLGDYGYLKDDY
WT	T1	ARGGYGD
WT	T1	ARGADGYLYYFDY
WT	T1	ARAYYGNLWFAY
WT	T1	ARGGNGLFAY
WT	T1	TRDQGITTALPYFDY
WT	T1	ARPPGCDVGFAY
WT	T1	ARHRVLRLFAY
WT	T1	AELSGYYFDY
WT	T1	ARDGYVAWFAY
WT	T1	ARGGLYYGNIYAMDY
WT	T1	ARHLTTAGAY
WT	T1	ARGGVYYFDY
WT	T1	AREGAFTTAIYWYFDV
WT	T1	AMGFPYAMDY
WT	T1	ARQGLSTMITTGYAMDY
WT	T1	ARRSTMITTVFAY
WT	T1	ARRITTVVATRAMDY
WT	T1	ARLGWLLPSWFAY
WT	T1	ARQGFITTVVPSFDY
WT	T1	ARALYGFYAMDY
WT	T1	AITTVVEGALAMDY
WT	T1	ARSITTVVDY
WT	T1	ARHLITTVVAPFAY
WT	T1	ARGPTMIIAMDY
WT	T1	ARLGGAY
WT	T1	ASVGDY
WT	T1	ARQYY

WT	T2	ARRAAY
WT	T2	ASNWDY
WT	T2	ARQRGS
WT	T2	ARHRYD
WT	T2	ARRRYDSREMDY
WT	T2	AREDYEKVDY
WT	T2	ARDDYYGY
WT	T2	ATNWDLGY
WT	T2	ARRSFDY
WT	T2	ARQDTFDY
WT	T2	ARENGLAY
WT	T2	ARHDYDWYFDV
WT	T2	ARRDGNSWFAY
WT	T2	ARQGDYFDY
WT	T2	ARSDFDY
WT	T2	ARREDYDGYAMDY
WT	T2	ARGGNRYDKEAYYFDY
WT	T2	ARHSDYGNWFAY
WT	T2	ARQGIRRNHSRSFRYFDV
WT	T2	AREDYYGSSEYFDY
WT	T2	ARQGYDGFAY
WT	T2	ARWEYGNYFDY
WT	T2	ARHGMDY
WT	T2	ARHHHGNYYAMDY
WT	T2	ARHNWGSSNDNYAMDY
WT	T2	AREYGNYY
WT	T2	ARHGRHYYAMDY
WT	T2	ARKDYGSSYFDY
WT	T2	ARHYYYGSNFDY
WT	T2	ARPTDWDAY
WT	T2	ARGGNYRTWFAY
WT	T2	ARDYGSSYEFAY
WT	T2	ARHGTGNFDY
WT	T2	ARDRGGTYWYFDV
WT	T2	ARDGDGNYGGYYFDY
WT	T2	ARDYYGSRGTMDY
WT	T2	ARDRGTGTGFAY
WT	T2	ARHYYGYYFDY
WT	T2	ARRNWADYAMDY
WT	T2	ARHSNYYAMDY
WT	T2	ARHGNGNFGDY
WT	T2	ARDGNYYYAMDY
WT	T2	ARGNYGPWFAY
WT	T2	ARDKSGYAMDY
WT	T2	ARGPYDGYYGPADY
WT	T2	ACHYYGSSFDY
WT	T2	ARSYGNYDYYAMDY

WT	T2	ARRDYGSSSYAMDY
WT	T2	ARPDYYGSSYGFAY
WT	T2	ARGDYYGSSPFAY
WT	T2	ARDYYYGSSSYWYFDV
WT	T2	ARRGGYGEYYAMDY
WT	T2	ARRGIYYDYDGWFAY
WT	T2	ARNYYGSSYDYAMDY
WT	T2	ARSGGKDYYAMDY
WT	T2	ARDDYGFFAY
WT	T2	ARGGGNYWYFDV
WT	T2	ARHDYGSSSAWFAY
WT	T2	ARHGGSSWGFDV
WT	T2	ARWDGNYVPMDY
WT	T2	ARHELSWFAY
WT	T2	ARDGSSSYYYAMDY
WT	T2	TRDQDSSGYVYAMDY
WT	T2	AREGGDYYGVWYFDV
WT	T2	ARGGNYYAMDY
WT	T2	ARDRGITTDAMDY
WT	T2	TRGGYYGSTYFDY
WT	T2	ARHYYGSSPLDYAMDY
WT	T2	ARGDYGSSYEVYYAMDY
WT	T2	ARQGNTTVGLDY
WT	T2	ARSGGKGAMDY
WT	T2	ARGWFGD
WT	T2	ARFDPYYYAMDY
WT	T2	ARGGTGTGLDY
WT	T2	ARGGFAY
WT	T2	ARGYDYDVGLGWGDY
WT	T2	ARGYGNYLYAMDY
WT	T2	ARLYDGYYGAMDY
WT	T2	ARHVLKDYAMDY
WT	T2	ARGEIYYDY
WT	T2	ARPPISLSDVPYWYFDV
WT	T2	ARAGDAMDY
WT	T2	ATTATLSYWYFDV
WT	T2	ARGGITTSWFAY
WT	T2	EATMITTDYAMDY
WT	T2	ARGGLGHAMDY
WT	T2	ARLVIHYYGRYAMDY
WT	T2	ARDGAVVAKGSFAY
WT	T2	ARGVSFQAWFAY
WT	T2	VRLAYYGFAY
WT	T2	ARITTVVPSYWYFDV
WT	T2	ARVPYGFYAMDY
WT	T2	ARPVGTGY
WT	T2	ARMITDAMDY

WT	T2	ASGWLLGAMDY
WT	T2	ARLLDY
WT	FO	ARNGAY
WT	FO	ARNGYRYEERGFDY
WT	FO	ARHPEEGLDY
WT	FO	ARHPEEGLDY
WT	FO	ARQRYYGNYDIDY
WT	FO	ARDHYRYGFAY
WT	FO	ARHRANWDY
WT	FO	AREDDYDGYYFDY
WT	FO	ARRSYGNYDY
WT	FO	AREDYGPFDY
WT	FO	ARPDYDGFAY
WT	FO	ARPNWDGFAY
WT	FO	ARHDRGDYYAMDY
WT	FO	ARQDYYGSNYDWYFDV
WT	FO	ARRGYDGYRSSFDV
WT	FO	ARRDGYYWYFDV
WT	FO	ARSGDDYGPQFAY
WT	FO	ARHGWDYFDY
WT	FO	ARDQGYGYAP
WT	FO	ARLGEREPNYFDY
WT	FO	ARHGNSRAMDY
WT	FO	ARHKENGYYGAMDY
WT	FO	AREGYGNYFDY
WT	FO	ARGRDGSSFDY
WT	FO	ARDYGKDYAMDY
WT	FO	ARTYDYDGPYFDY
WT	FO	ARHGGYNYFDY
WT	FO	ARGQGYYDNYDAMDY
WT	FO	ARHDYGSSFDY
WT	FO	ARQGPYYFDY
WT	FO	ARRQMYGNYGFDY
WT	FO	ARHEEDHYGSSYVFDY
WT	FO	ARRYDGAMDY
WT	FO	ARRAGDYFDY
WT	FO	ARHGGNYPYWYFDV
WT	FO	ARESWGNYSYYFDY
WT	FO	ARDRDYYGSSYYAMDY
WT	FO	ARQRGSWWDRAYYYAMDY
WT	FO	ATPGTPRGYYFDY
WT	FO	AREGYDYDGAWFAY
WT	FO	ARGGNPDY
WT	FO	ARGGNYYFDY
WT	FO	ANDYEGAMDY
WT	FO	ARLDYRYDGDAMDY
WT	FO	ARPGYGNYGYFDV

WT	FO	ARHRFDGYYPYYYAMDY
WT	FO	ARQGYYYGSSYFDY
WT	FO	ARHLYDYYFDY
WT	FO	ARHVNYGYDWTWFAY
WT	FO	ARGHYYGSSSFAY
WT	FO	TREGYYGSSGFAY
WT	FO	ARQRGYYGSSFSYWYFDV
WT	FO	ARHFYDGYSFAY
WT	FO	ARHEAYLNMDWFAY
WT	FO	ARRADYGSSWAMDY
WT	FO	ARHRAYGNFYYYAMDY
WT	FO	ARWGYRGAMDY
WT	FO	ARSYYYGSSYGFAY
WT	FO	ARQDYYGVDAMDY
WT	FO	ASLVYYRYDEGDY
WT	FO	ARQGYSSYAMDY
WT	FO	AREGR*LPYYAMDY
WT	FO	ARDGYAMDY
WT	FO	ARDANSLLRLRSRYFDV
WT	FO	ARGSSYGYFDY
WT	FO	ARLGGNYGEFAY
WT	FO	ARYGKGYCFYY
WT	FO	ARGEGSYAFDY
WT	FO	ARHIGNYFDY
WT	FO	ARHGNPVYYAMDY
WT	FO	ARGGYYVDWYFDV
WT	FO	ARSDGYYLMDY
WT	FO	ARTGFDY
WT	FO	ARHYYGYVYYYAMDY
WT	FO	ARTSPMKGAMDY
WT	FO	ARLFYSQWDFDV
WT	FO	ARHTAYYAMDY
WT	FO	ARRYGSSYLAMDY
WT	FO	TRDGGLGPFDY
WT	FO	ARGYYDVWGYYAMDY
WT	FO	ARTGYYGNYLAWFAY
WT	FO	ARGELLWSYKGFAY
WT	FO	ARSGGYYAMDY
WT	FO	ARDGGLRPLYYAMDY
WT	FO	ARRGGFYAMDY
WT	FO	ARQELRVGSSFFAY
WT	FO	ARHGGVPFAY
WT	FO	ARHLTGIDY
WT	FO	ARSEGLRFAYYAMDY
WT	FO	ARSFGGGTWFAY
WT	FO	ARAPEGSMITTWGFAY
WT	FO	ARGLYYRYVYAMDY

WT	FO	ARIYYGYGFDY
WT	FO	AREGWLLRAMDY
WT	FO	ARGGEVRVYAMDY
WT	FO	ARIGDGSSLSYAMDY
WT	FO	AREVRLAMDY
WT	FO	ARRSISLYAMDY
WT	FO	ARGPTMITMDY
WT	FO	ARQTGLLPFFDY
WT	FO	ARRITTVVATRAMDY
WT	FO	ARRIGMAFAY
WT	FO	ARQITTVVADYAMDY
WT	FO	ARRITAVVATRAMDY
WT	FO	ARGGLLPGAMDY
WT	FO	ARHITTVVAYYAMDY
WT	FO	ARLFITTATKAMDY
WT	FO	ARVSTMITTFDY
WT	FO	ARLGLLRAMDY
WT	FO	ARLITTVVATRAMDY
WT	FO	AREAY
WT	MZ	ARRRGDY
WT	MZ	ARENDYDEGFAY
WT	MZ	ARKDYDYFDY
WT	MZ	ARHDYDYLDY
WT	MZ	ARRWDYFDY
WT	MZ	ARQDGFDY
WT	MZ	TSYYDQRGFAY
WT	MZ	ARDYFDY
WT	MZ	ARHEDGYYPY
WT	MZ	AREEDGSLDY
WT	MZ	ARQGDYDYFDY
WT	MZ	ARQRDGSSFDY
WT	MZ	ARHHGNGFAY
WT	MZ	ARHEEYGNYEAMDY
WT	MZ	ASDYYFDY
WT	MZ	ARRTGYHFDY
WT	MZ	ARHSPYDYPDY
WT	MZ	ARTGTRDFDY
WT	MZ	ARHMDYDYDV
WT	MZ	ANPYYRYDDAMDY
WT	MZ	ARDGQIRGRDYFDY
WT	MZ	ARDDGYGWAY
WT	MZ	ARDGNYWYFDV
WT	MZ	ARGRDYYGSSSDFDY
WT	MZ	ARQTSFDY
WT	MZ	ARPEDYGGPYYFDY
WT	MZ	ARREVRNAMDY
WT	MZ	AREGWGNYRAMDY

WT	MZ	ARGYDYYFDY
WT	MZ	ARHDYGYRGYAMDY
WT	MZ	ARHYYGYVDY
WT	MZ	ARSPYGNYVDY
WT	MZ	AVEYGTFDY
WT	MZ	ARHGLRHYFDY
WT	MZ	ARQGGYGNWYFDV
WT	MZ	ARQGRDVYFDY
WT	MZ	ARRYGGSMDY
WT	MZ	ARSYYGNLDY
WT	MZ	ARRDGSAIDY
WT	MZ	AKGGNYGY
WT	MZ	ARQGGNDY
WT	MZ	ARQDYGSSYGWYFDV
WT	MZ	ARGGNYYFDY
WT	MZ	ARHGNYAMDY
WT	MZ	ARGDRYYVRGDYYAMDY
WT	MZ	AKYGNYNSYAMDY
WT	MZ	ARDYGNSYAMDY
WT	MZ	ARSYYYGSSYTRYWYFYV
WT	MZ	ARTYYYGSSYDYFDY
WT	MZ	ASHHLWDGYLAY
WT	MZ	AYHYYGSSYWYFDV
WT	MZ	ARVRQRAMDY
WT	MZ	ARQGGSPRGYYAMDY
WT	MZ	ARHDYYGSSYYYAMDY
WT	MZ	ARHSSSGYDY
WT	MZ	TRDQGGNYVFDY
WT	MZ	ARSELRTYYFDY
WT	MZ	ARHNGNYAAMDY
WT	MZ	ARIDMRRYPY
WT	MZ	ARHAGNYEGYYYAMDY
WT	MZ	ARGGETTATRSPFAY
WT	MZ	ARGGYYGNGFAY
WT	MZ	ARGGGKGMDY
WT	MZ	ARGARYFDV
WT	MZ	ASPYGSRSPGAMDY
WT	MZ	ASYYGSSYYFDY
WT	MZ	ARAPYGNYGYFDV
WT	MZ	ARQAYDYAWFAY
WT	MZ	ARYYGNYGYAMDY
WT	MZ	ARQGGYYGSFHMDY
WT	MZ	ARQGGYYGSFHMGY
WT	MZ	ARGRGYAMDY
WT	MZ	ARYGNYAMDY
WT	MZ	ARQGGYYAMDY
WT	MZ	ANYGSSYGY

WT	MZ	ARKDYGSFYAMDY
WT	MZ	ARGGYYGSGYGFVY
WT	MZ	ARHAMDY
WT	MZ	AIYYYGSSYAMDY
WT	MZ	ARRAARATFAY
WT	MZ	ARWGLRYFDY
WT	MZ	ARLGDYTGYYFDY
WT	MZ	ARRITSYYFDY
WT	MZ	ARVSYGNYGGWYFDV
WT	MZ	ARAYYGSPFDY
WT	MZ	ARSGTGFDY
WT	MZ	ARMGTTARYFDV
WT	MZ	ARGGFAY
WT	MZ	ARGGSAY
WT	MZ	ARVYGSSYDYAMDY
WT	MZ	ARGVYGSSYWYFDV
WT	MZ	ARQLTGTGAY
WT	MZ	ARATGTDAMDY
WT	MZ	ARQDTTVVAHFDY
WT	MZ	ARHGDLTMITGGWYFDV
WT	MZ	ARHGRSIGTATVYFDY
WT	MZ	ARLGLDYYGYLSY
WT	MZ	ARGGTVVAKDYAMDY
WT	MZ	ARHVYGFPDY
WT	MZ	ARHVITTGYAMDY
WT	MZ	ARQGTTVVTDY
WT	MZ	ARQEGITTVVFDY
WT	MZ	ARHLMITTSYAMDY
WT	MZ	ARGLSTVVDYFDY
WT	MZ	AREVLRLFDY
WT	MZ	ARHVTTVVAPSFDY
WT	MZ	ARSLLRLRAY
WT	MZ	ARVMIRAWFAY
WT	MZ	ARAMITTFYAMDY
WT	MZ	ASLITTALDY
